# Epidemiological investigation of perinatal depression among pregnant and postpartum women: findings from a cross-sectional survey in the Philippines

**DOI:** 10.1136/bmjopen-2025-109079

**Published:** 2026-02-18

**Authors:** Joycelyn Abiog Filoteo, Joemer Calderon Maravilla, Jose Edwardo Mamaat, Arbie Diane Flores, Ana Nelia Jumamil, Reinalyn Lim Cardenas, Wilfredo Quijencio, Mary Ann Bayani, Nemencio Santos, Joyce Lisa Acena, Anna Liza Alfonso, Mayla Rivera, Rebecca Guariño, Rachelle Sarmiento, Vicki Flenady, Frances M Boyle, Siobhan A Loughnan, Alma Trinidad Taragua

**Affiliations:** 1 Department of Nursing, Institute of Health Sciences and Nursing, Far Eastern University, Manila, Philippines; 2 School of Public Health, The University of Queensland, Herston, Queensland, Australia; 3 Queensland Centre for Mental Health Research, Wacol, Queensland, Australia; 4 Filipino Nursing Diaspora Network, Sydney, New South Wales, Australia; 5 Department of Psychology, Institute of Arts and Sciences, Far Eastern University, Manila, Philippines; 6 JPMC College of Health Sciences, Bandar Seri Begawan, Brunei Darussalam; 7 NHMRC Centre of Research Excellence in Stillbirth, Mater Research Institute, The University of Queensland, South Brisbane, Queensland, Australia; 8 Institute for Social Science Research, The University of Queensland, Indooroopilly, Queensland, Australia

**Keywords:** MENTAL HEALTH, Depression, Postpartum, EPIDEMIOLOGIC STUDIES, PUBLIC HEALTH

## Abstract

**Abstract:**

**Objective:**

This study investigated perinatal depressive symptoms among pregnant and postpartum Filipino women.

**Design:**

Cross-sectional survey.

**Setting:**

The Philippines.

**Participants:**

Participants were recruited online and face-to-face from maternal care facilities.

**Primary outcome measure:**

Perinatal depressive symptoms were assessed using the Edinburgh Postnatal Depression Scale (EPDS) score, with prevalence calculated based on ≥13 cut-off, indicating clinically significant symptoms of depression. Patterns of depressive symptoms were examined by demographics, perinatal time period and other obstetric information using adjusted regression coefficients (a*b*) and risk ratios (aRR).

**Results:**

A total of 856 women participated in the study, comprising 356 pregnant and 500 postpartum women. EPDS scores were, on average, similar between pregnant (14.4) and postpartum women (14.1). Using the locally validated cut-off of ≥13 revealed that 69.1% of pregnant and 62.0% of postpartum women reported clinically significant depressive symptoms. Consistent EPDS scores and prevalence were observed across pregnancy trimesters and within 12 months postpartum and beyond. Women who received adequate prenatal care were less likely to experience antenatal (a*b*=−1.59, 95% CI −3.13 to −0.05) and postpartum (a*b*=−1.30, 95% CI −2.48 to −0.12) depressive symptoms. Postpartum EPDS scores and depressive symptom prevalence (EPDS score ≥13) were higher among 18–24-year olds (a*b*=1.96, 95% CI 0.30 to 3.61; aRR=1.23, 95% CI 1.03 to 1.47) and single mothers (a*b*=3.46, 95% CI 0.22 to 6.71; aRR=1.42, 95% CI 1.07 to 1.90), compared with older and married mothers, respectively.

**Conclusions:**

At least 60% Filipino mothers experienced clinically significant perinatal depressive symptoms, which exceeds the established global average of 25%. Younger and single postpartum women were at greater risk, while pregnant and postpartum women who attended adequate prenatal visits were less likely to report depressive symptoms. Our study underscores the need for further research to uncover the true burden of poor perinatal mental health and calls for targeted early interventions and integrative public health strategies to support at-risk mothers, particularly those from socially disadvantaged backgrounds.

**STRENGTHS AND LIMITATIONS OF THIS STUDY**

- This study pioneers the investigation of perinatal depressive symptoms during and after the COVID-19 pandemic in the Philippines, examining perinatal depression prevalence from a large-scale online and facility-based sample across the country.
- This study recruited a non-probability sample of pregnant and postpartum women; hence, results from this study should be interpreted with caution.
- While considered a gold standard in screening for perinatal depressive symptoms, the Edinburgh Postnatal Depression Scale is not a diagnostic instrument and is unable to assess functional impairment related to perinatal depressive symptoms.

## Background

 The perinatal period represents a critical window for the health and well-being of mothers and their children. The rapid physiological, social and emotional changes have profound implications for maternal well-being as well as fetal development and child health outcomes. The biopsychosocial changes and parenting-related stress predispose mothers to depressive episodes, which may onset from conception and may last for months or years after birth.^1^ If not addressed, maternal depression during pregnancy can result in adverse obstetric outcomes, poor health-seeking behaviour and negative long-term impacts on the socio-emotional development of children.^2^

Perinatal depression or maternal depression is characterised by non-psychotic depressive symptoms such as low mood, anhedonia, inappropriate guilt and suicidal behaviours.^1^ Symptoms range from mild to severe and may occur during pregnancy and/or after childbirth,^2^ commonly assessed using self-reported screening instruments such as the Edinburgh Postnatal Depression Scale (EPDS), Patient Health Questionnaire and Beck Depression Inventory.^3^ Previous studies in high-income countries revealed that prevalence estimates range from 10.0% to 20.0%.^4^ Perinatal depression prevalence in low and middle-income countries (LMICs) was at least two times higher compared with prevalence in high-income countries,^4^ with a recent global prevalence of 24.7% pooled from 51 LMICs.^3^ Depression prevalence in the antepartum period (26.3%) was slightly higher than in the postpartum period (23.1%).^3^

The growing global evidence on perinatal depression in LMICs highlighted wide country variations in prevalence in Asia and the Pacific due to cultural diversity and health system differences within the region.^2^,^4^ Despite good data representation of countries within this region, there remains a data gap in the Philippines. The mental health burden among Filipino women remains poorly documented, and studies on perinatal mental health are particularly scarce. The only national data on women’s mental health comes from the 2021 Philippine National Survey on Mental Health and Well-being. This survey showed that the lifetime and past 12-month prevalence of any mental disorder among women aged 18 years or older were 12.7% and 9.63%, respectively.^5^ The survey also indicated that 1.61% and 1.66% of women experienced major depressive disorder and a major depressive episode in the past 12 months. To date, no studies have assessed antenatal depression, and only three studies have examined postpartum depression; each based on small facility-based samples from a tertiary hospital^6 7^ and five birthing facilities.^8^

Understanding the burden of perinatal depression in the Philippines is essential, given the complex interplay of socio-economic and cultural determinants influencing perinatal mental health. Despite its significant implications, perinatal depression remains largely unrecognised and undertreated in the country, reflecting the absence of comprehensive policies and clinical guidelines for supportive perinatal care and mental health. This study aims to describe depressive symptoms and estimate the prevalence of antenatal and postpartum depression among Filipino mothers. Using a large-scale sample of pregnant and postpartum mothers during the latter phase of the pandemic, this research provides empirical data to inform the development of locally relevant and globally aligned maternal mental health policies and interventions.

## Methods

### Study design

A cross-sectional survey was developed in partnership with the COCOON Global Collaboration, coordinated by Australia’s National Health and Medical Research Council (NHMRC) Centre of Research Excellence in Stillbirth (Stillbirth CRE) in Brisbane, Australia. COCOON’s published global protocol discussed the countries involved in the survey, the overarching survey methodology and measures, and the anticipated survey administration period.^9^

### Patient and public involvement

Service providers and government stakeholders were involved in the design and conduct of this study. Our research team in the Philippines conducted a knowledge transfer workshop to inform the priority areas and key measures of the survey as well as approaches in disseminating study results.

### Sample and recruitment

Aligned with the published protocol,^9^ a non-probability sample of currently pregnant women or those who had given birth on or after 30 January 2020, and aged 18 years or older, was recruited online using Facebook as well as face-to-face through maternal care facilities. In this current study, we excluded postpartum women who experienced miscarriage, stillbirth and neonatal death in their last pregnancy due to their psychological experiences and planned separate analysis of outcomes for this group.^9^

Facebook is considered the most used social media platform by Filipinos 18 years or older and Facebook ads have been documented to reach 93.1% of users in the Philippines.^10^ We deployed paid Facebook ads weekly for 6 weeks from March 2024 to April 2024. The ads enabled interested users to directly inform the research team of their interest in participating using Facebook Messenger. Apart from screening for eligibility, we verified their account name, date of birth, age of gestation and date of delivery (if applicable) to ensure that users were not operated by bots or fraudulent users. We increased the reach of recruitment by approaching eligible pregnant and postpartum women face-to-face in seven maternal care facilities, including four community health centres and three birthing clinics across Metro Manila and Rizal Province, from November 2023 to May 2024.

### Survey development and procedure

The survey was initially developed at the beginning of the COVID-19 pandemic as part of a multicountry collaboration to examine parent psychosocial well-being and experiences of maternity, neonatal and perinatal bereavement care. In the Philippines, the survey was further adapted in the local setting in 2022–2023. Of the eight surveys developed by the COCOON Global Collaboration, Surveys A and B (as per the protocol)^9^ were deployed to assess depressive symptoms among pregnant and postpartum women in the Philippines. The self-administered questionnaire in both surveys included chronologically sequenced modules on their current or immediately past pregnancy, psychosocial well-being and sociodemographic characteristics.^9^ The questionnaire as well as the participant information sheet and consent form were translated into Tagalog-English and pilot-tested among 10 pregnant and postpartum mothers. Eligible and verified participants online were provided with a survey link to access the information sheet, consent form and the questionnaire in English and in Tagalog-English. The data collection lasted for 6 months, between November 2024 and May 2024.

The study used the following measures:

*Obstetric profile*. Pregnant participants were asked about their current gestational age (AOG) in weeks. For postpartum participants, AOG was reported in reference to the delivery of their latest pregnancy. Both participant groups were also asked if their current or latest pregnancy was their first pregnancy. Adequacy of their antenatal visits was assessed based on their number of antenatal visits and AOG using WHO guidelines.^11^ They were also asked about the type of facility where they accessed pregnancy care most of the time.

*Perinatal depressive symptoms*. The EPDS,^12^ a well-established 10-item scale, was used to assess symptoms of depression in pregnant and postpartum women. Each item is rated on a 4-point scale, and total scores can range from 0 to 30. In this study, EPDS has a Cronbach’s alpha of 0.851 among pregnant women and 0.860 among postpartum women. All participants completed every item of the EPDS.

While most existing studies limit their samples to women within 12 months postpartum,^3 13^ our study adopted a broader temporal scope by administering EPDS to pregnant and postpartum women regardless of time since birth. This approach is informed by recent findings from prospective studies in more than 20 LMICs and high-income countries (HICs), which reported persistent perinatal depressive symptoms extending from pregnancy up to 36+ months postpartum.^14^,^17^

*Demographic characteristics*. The current age was derived using the date of birth and was further grouped into three categories: 18–24-year olds, 25–34-year olds and 35 years or older. Highest educational attainment and marital status were also asked among participants. Urbanicity was determined using the municipality, city or province reported by the participants and classifications established by the Philippine Statistics Authority.^18^ The location of the participants was based on the three main island groups of the Philippines: Luzon, Visayas and Mindanao, with the National Capital Region (located in Luzon) treated as a separate category as the country’s capital.

Participants were able to skip questions as they wished. The distribution of non-response is shown in online supplemental table S1. At survey completion, participants were asked to voluntarily provide their contact details for a follow-up survey and a qualitative interview, which are outside the scope of this current study. Identifying information was stored separately from survey responses.

### Verification and data preparation

Participants were asked to inform the research team through Facebook Messenger on survey completion. The research team then verified the responses by checking the date of birth, gestational age and date of delivery (if applicable) using the information provided during the screening. Mismatched responses were excluded. Participants who completed the survey in under 10 min were asked to retake it based on the minimum time of completion identified during pilot testing. Responses from those who were not willing to retake or those who continued to complete the survey quickly were rejected. Those who were recruited in person were asked to sign informed consent and self-complete the survey using a computer tablet. Participant information was verified using the approach discussed above. Participants who completed the survey were financially compensated for their time.

The final sample included participants who had completed at least the Psychosocial Health module, which contains the EPDS. Because this module appears in the latter part of the questionnaire, participants who partially completed the survey up to this point had only skipped the sociodemographic set of questions.

### Data analysis

All analyses were conducted using Stata version 18.^19^ Descriptive statistics were used to summarise the characteristics of both pregnant and postpartum women. The prevalence of clinically significant perinatal depressive symptoms (also referred to as perinatal depression in this paper) was determined based on the cut-off score of ≥13, which was recommended by a recent validation study in the Philippines.^20^ Statistical differences were assessed using 95% CIs. Overlapping 95% CIs suggest that the differences between estimates are not statistically significant.

Multivariable linear and negative binomial regression analyses were conducted to examine epidemiological patterns of depressive symptoms, using the EPDS as a continuous score and a binary variable, respectively. Regression analyses were used to examine the trend of depressive symptoms and prevalence by perinatal time period (combined pregnant and postpartum sample) and by timing of birth (postpartum women only). Analysis by perinatal time period was performed using a time variable generated based on AOG among pregnant women and months after birth among postpartum women (time variable categories: first, second and third trimesters, 0–12 months, 13–24 months and 25+ months postpartum). Separate regression analysis was conducted using the timing of birth variable, which has two categories: during the pandemic and after the pandemic (from July 2023 onward). This split reflects the lifting of the COVID-19 public health emergency status by the Philippine government in July 2023.^21^ All analyses were adjusted for demographic characteristics and obstetric profile.

## Findings

### Sample characteristics

A total of 856 women who participated in the survey were included in the final sample, which comprised 356 pregnant women and 500 postpartum women, with 98.6% and 90.7% response rates (see online supplemental figure S1). The majority of pregnant (n=297, 83.4%) and postpartum women (n=409, 81.8%) in the final sample were recruited online, noting that the mode of recruitment was not statistically different between participant types (X^2^=0.38, p=0.537).

As shown in table 1, both pregnant and postpartum women were predominantly aged 25–34 years and mostly lived in urban areas. More postpartum women had a secondary education or lower (55.9%), compared with pregnant women (48.0%). Most participants were either married and living with their spouses or living with a partner. A similar proportion of pregnant women (31.9%) and postpartum women (30.5%) reported that their current or most recent pregnancy was their first.

**Table 1:** Characteristics of the final sample

	Pregnant women % (n)	Postpartum women % (n)
N	**356**	**500**
SOCIO-DEMOGRAPHICS		
Current age (in years)		
Mean (SD, IQR)	29.5 (6.0, 24.9–34.0)	30.4 (6.0, 26.4–33.9)
18–24	25.3% (90)	17.0% (85)
25–34	57.0% (203)	61.4% (307)
35 or older	17.7% (63)	21.6% (108)
Highest educational attainment		
Secondary or lower	48.0% (158)	55.9% (272)
University or higher	52.0% (171)	44.2% (215)
Current relationship status		
Married and living with spouse	47.1% (161)	50.1% (244)
Not married but living with a partner	41.8% (143)	40% (195)
In a relationship but living separately	5.9% (20)	1.9% (9)
Married but not living with spouse	3.2% (11)	3.1% (15)
Single	1.8% (6)	3.3% (16)
Other	NR	1.6% (8)
Urbanicity		
Rural	25.1% (88)	19.9% (98)
Urban	74.9% (263)	80.1% (394)
Location		
Luzon	64.2% (226)	69.6% (344)
National Capital Region	23.6% (83)	22.5% (111)
Visayas	7.1% (25)	5.3% (26)
Mindanao	5.1% (18)	2.6% (13)
OBSTETRIC PROFILE		
First pregnancy*		
Yes	31.9% (109)	30.5% (149)
No	68.1% (233)	69.5% (340)
Main health facility for pregnancy care†		
Government clinic	30.6% (107)	32.4% (160)
Government hospital	29.7% (104)	29% (143)
Private clinic (or ‘lying-in’ clinic)	26.3% (92)	25.5% (126)
Private hospital	12.0% (42)	12.2% (60)
Other	NR	NR
Adequate prenatal visits attended†		
Yes	74.2% (257)	57.1% (269)
No	25.7% (89)	42.9% (202)
Gestational age†		
Mean (SD, IQR)	21.3 (10.4, 12.0–30.0)	38.1 (1.9, 37.0–39.0)
Number of months since birth*		
Mean (SD, IQR)	n/a	25.8 (16.0, 11.0–41.0)
Perinatal time period at the time of the survey†		
First trimester	25.6% (91)	n/a
Second trimester	45.8% (163)	n/a
Third trimester	28.7% (102)	n/a
0–12 months postpartum	n/a	25.8% (129)
13–24 months postpartum	n/a	22.2% (111)
25+months postpartum	n/a	52.0% (260)
Timing of birth‡		
During the pandemic	n/a	78.4% (392)
After the pandemic	n/a	21.6% (108)
EPDS score at the time of the survey		
Mean (SD, IQR)	13.6 (4.5, 11.0–17.0)	13.7 (4.8, 10.0–17.0)

Frequencies do not add up to N due to non-response.

* For postpartum women, they were asked if their recent pregnancy (ie, reference pregnancy) was their first pregnancy.

† For postpartum women, this pertains to the reference pregnancy.

‡ July 2023 was announced as the end of the pandemic in the Philippines and was used as the reference point; for pregnant women, this is not applicable since all participated in the survey after the pandemic.

EPDS, Edinburgh Postnatal Depression Scale; n/a, not applicable; NR, estimates not reported due to denominator≤5.

Government facilities were the main sources of pregnancy care for both groups, with 74.2% of pregnant and 57.1% of postpartum women found to have adequate prenatal visits. The mean AOG of pregnant women was 21.3 weeks, while for postpartum women was 38.1 weeks. At the time of the survey, the majority of the pregnant women was in their second trimester (45.8%), while almost half of the postpartum women were 0–24 months postpartum (48.0%).

### Perinatal depressive symptoms

The mean EPDS score was similar between pregnant (mean=14.4, SD=5.7, range=0–29) and postpartum women (mean=14.1, SD=6.2, range=0–30). The prevalence of antenatal depression was 69.1% (95% CI 64.1% to 73.7%) while postpartum depression was 62.0% (95% CI 57.7% to 66.2%).

Comparable mean EPDS scores and prevalence across trimesters among pregnant women and time since birth among postpartum mothers were observed (see table 2). This finding is consistent with the time effects observed in multivariate regression analyses, where depressive symptoms are treated as a score outcome (adjusted coefficient (a*b*)=−0.01, 95% CI −0.45 to 0.43) and a binary outcome (adjusted risk ratio (aRR)=0.98, 95% CI 0.92 to 1.03).

**Table 2:** Mean EPDS score and prevalence of perinatal depression across the perinatal period and by timing of birth

Time variable	N	Mean EPDS score (95% CI)	Prevalence (95% CI)
Perinatal time period at the time of the survey			
First trimester	91	13.8 (12.6 to 15.0)	65.9 (55.6 to 74.9)
Second trimester	163	14.4 (13.5 to 15.2)	66.3 (58.7 to 73.1)
Third trimester	102	14.8 (13.8 to 15.9)	76.5 (67.3 to 83.7)
0–12 months postpartum	129	14.0 (13.1 to 15.0)	60.5 (51.8 to 68.5)
13–24 months postpartum	111	13.6 (12.5 to 14.8)	55.9 (46.5 to 64.8)
25+months postpartum	260	14.4 (13.6 to 15.2)	65.4 (59.4 to 70.9)
Time effect		b=0.01 (−0.22 to 0.23) Adjusted b=−0.01* (−0.45 to 0.43)	RR=0.99 (0.96 to 1.01) Adjusted RR=0.98† (0.92 to 1.03)
Timing of birth (among postpartum only)‡			
During the pandemic	392	14.3 (13.7 to 14.9)	63.3 (58.4 to 67.9)
After the pandemic	108	13.6 (12.5 to 14.7)	66.4 (61.9 to 70.5)
Time effect		b=0.67 (−0.65 to 1.98) Adjusted b=0.39* (−1.56 to 2.33)	RR=1.10 (0.92 to 1.32) Adjusted RR=1.01† (0.79 to 1.31)

Prevalence was based on ≥13 cut-off score.

* Adjusted regression coefficient from multivariable linear regression, adjusted for highest educational attainment, urbanicity, first pregnancy status, type of health facility during pregnancy, adequacy of prenatal visits and current gestational age.

† Adjusted risk ratio from multivariable negative binomial regression, adjusted for highest educational attainment, urbanicity, first pregnancy status, type of health facility during pregnancy, adequacy of prenatal visits and gestational age since birth of reference pregnancy.

‡ July 2023 was announced as the end of the pandemic in the Philippines and was used as the reference point; for pregnant women, this is not applicable since all participated in the survey after the pandemic.

EPDS, Edinburgh Postnatal Depression Scale; N, sample size.

The prevalence of clinically significant postpartum depressive symptoms was not statistically different between those who gave birth during the pandemic (63.3%, 95% CI 58.4% to 67.9%) and after the pandemic (66.4%, 95% CI 61.9% to 70.5%). This was confirmed by multivariable regression (aRR=1.01, 95% CI 0.79 to 1.32).

### Patterns by demographic characteristics and obstetric profile

Among pregnant women, no difference in depressive symptoms and prevalence was observed by sociodemographic characteristics and pregnancy-related information, except for adequacy of prenatal visits (online supplemental table S2). Pregnant women who had adequate prenatal visits showed lower EPDS scores (a*b*=−1.59, 95% CI –3.13 to −0.05). In terms of prevalence, regression analysis showed a significant crude RR (0.80, 95% CI 0.71 to 0.92) but not after adjustment.

Among postpartum women, statistically significant associations were found for age, relationship status and adequacy of prenatal visits (see table 3; see online supplemental table S3 for full regression results).

**Table 3:** Depression among postpartum mothers by age, relationship status and adequacy of prenatal visits

	EPDS score			Prevalence of postpartum depression		
	Mean (SD)	*b* (95% CI)	a*b* (95% CI)*	% (n)	RR (95% CI)	aRR (95% CI)†
Age						
18–24	15.8 (5.5)	1.32 (−0.14 to 2.77)	1.96 (0.30 to 3.61)	72.9% (62)	1.16 (0.99 to 1.36)	1.23 (1.03 to 1.47)
25–34	14.4 (6.2)		Ref	62.9% (193)	Ref	Ref
35 or older	12.0 (6.1)	−2.44 (−3.77 to −1.11)	−2.24 (−3.65 to −0.82)	50.9% (55)	0.81 (0.66 to 0.99)	0.86 (0.69 to 1.06)
Current relationship status						
Married and living with spouse	13.4 (6.1)	Ref	Ref	58.6% (143)	Ref	Ref
Not married but living with a partner	15.0 (6.0)	1.61 (0.46 to 2.76)	1.53 (0.33 to 2.73)	66.7% (130)	1.14 (0.98 to 1.31)	1.12 (0.96 to 1.31)
In a relationship but living separately	16.3 (6.1)	2.94 (−1.13 to 7.01)	3.08 (−1.19 to 7.35)	77.8% (7)	1.33 (0.92 to 1.91)	1.41 (0.99 to 2.03)
Married but not living with spouse	13.3 (5.9)	−0.06 (−3.25 to 3.13)	0.62 (−2.66 to 3.89)	60.0% (9)	1.02 (0.67 to 1.57)	1.07 (0.67 to 1.70)
Single	16.3 (7.9)	2.92 (−0.17 to 6.01)	3.46 (0.22 to 6.71)	75.0% (12)	1.28 (0.95 to 1.73)	1.42 (1.07 to 1.90)
Other	16.3 (5.1)	2.86 (−1.45 to 7.16)	1.08 (−3.81 to 5.97)	75.0% (6)	1.28 (0.85 to 1.94)	1.06 (0.64 to 1.74)
Adequate prenatal visits attended						
Yes	13.2 (6.3)	−1.78 (−2.89 to -0.67)	−1.30 (−2.48 to −0.12)	52.0% (148)	0.79 (0.69 to 0.91)	0.83 (0.72 to 0.97)
No	15.0 (5.8)	Ref	Ref	69.3% (140)	Ref	Ref

* Adjusted regression coefficient (a*b*) from multivariable linear regression, adjusted for highest educational attainment, urbanicity, first pregnancy status, type of health facility during pregnancy, adequacy of prenatal visits, gestational age since birth of reference pregnancy, number of months since birth of reference pregnancy.

† Adjusted risk ratio (aRR) from multivariable negative binomial regression, adjusted for highest educational attainment, urbanicity, first pregnancy status, type of health facility during pregnancy, adequacy of prenatal visits, gestational age since birth of reference pregnancy, number of months since birth of reference pregnancy.

%, prevalence based on ≥13 cut-off score; EPDS, Edinburgh Postnatal Depression Scale.

*Age*. EPDS scores were higher among 18–24-year olds but lower among 35-year olds or older compared with 25–34-year olds. This was confirmed through multivariable regression analyses: 18–24 (a*b*=1.96, 95% CI 0.30 to 3.61) and 35 or older (a*b*=−2.24, 95% CI −3.65 to −0.82).

*Relationship status*. Postpartum women who reported their relationship status as single (ie, not married and do not have a partner) showed significantly higher depression scores compared with married women who currently live with their spouse. This is consistent with results from regression analysis (a*b*=3.46, 95% CI 0.22 to 6.71). In terms of prevalence, single mothers were 42% more likely (aRR=1.42, 95% CI 1.07 to 1.90) to report clinically significant symptoms compared with married women who lived with their spouse. Postpartum women who were not married but living with a partner also reported slightly elevated symptom scores (a*b*=1.53, 95% CI 0.33 to 2.73).

*Adequacy of prenatal visits*. Those with adequate prenatal visits in their recent pregnancy reported lower EPDS scores (a*b*=−1.30, 95% CI –2.48 to −0.12) compared with those with inadequate visits. Adequate prenatal visits were also associated with a reduced risk of clinically significant depressive symptoms (aRR=0.83, 95% CI 0.72 to 0.97).

## Discussion

This study measured depressive symptoms among pregnant and postpartum women who had given birth since the pandemic in the Philippines. On average, depressive symptoms in both groups were clinically significant, indicating the need for further assessment and management. At least three in every five pregnant or postpartum Filipino women experienced clinically significant depressive symptoms. Women who received adequate prenatal care were less likely to experience antenatal and postpartum depressive symptoms. Differences in postpartum depressive symptoms were found between age groups and between relationship status, with increased likelihood of postpartum depression among 18–24-year olds and single mothers compared with those who are at least 25-years old and married, respectively.

To our knowledge, this is the first study in the Philippines to investigate the prevalence of antenatal and postpartum depression mainly from an online-based sample, which enabled reaching mothers across the country. Previous studies in the Philippines have also utilised convenience samples of postpartum women who gave birth within the first 8 weeks. For example, a prepandemic survey of 165 mothers who accessed maternal care from five rural health facilities showed lower postpartum depression prevalence estimates of 16.4% using an EPDS cut-off of ≥10.^8^ A hospital-based study of 61 postpartum women who gave birth during the pandemic also showed a lower prevalence of 32.8% (vs 54.2% found by our study among mothers 0–3 months postpartum, see online supplemental table S4) despite using a higher score threshold (EPDS score ≥13).^6^ As such, facility-based studies may be subjected to sampling bias, as participants who access healthcare services are more likely to exhibit better health-seeking behaviours compared with the general population. Results from a meta-analysis of 160 studies in LMICs conversely showed statistically similar prevalence estimates between community-based, population-based and facility-based studies.^3^ However, studies included in this review were mainly conducted before the pandemic and may not account for disparities in services and health behaviours between primary care and higher levels of care.

This study demonstrated the consistently high prevalence of clinically significant perinatal depressive symptoms among both pregnant and postpartum mothers. The similarity in prevalence between these groups aligns with findings from previous research, including an umbrella review of 12 meta-analyses, which reported comparable global prevalence of depressive symptoms across the perinatal period during the pandemic (antenatal depression=29.0% and postpartum depression=26%).^22^ However, the prevalence estimates found by our study are alarmingly higher compared with the global prevalence pooled by this umbrella review and the prepandemic prevalence in another review in Southeast Asia.^23^ Using the least (ie, ≥11) and the most conservative (ie, ≥13) validated cut-off scores, the prevalence in our study remained higher than the global average.

The differences in prevalence between our study and previous research may be explained by the profound impact of the pandemic on women’s mental health as well as the persistent gaps in perinatal mental health services in the Philippines. The Global Burden of Disease study reported a disproportionate rise in major depressive disorder (MDD) prevalence among women compared with men, with the Philippines experiencing the highest increase in MDD prevalence across Southeast Asia.^24^ A recent meta-analysis of 22 studies comparing postpartum depression prevalence before and during the pandemic found a significant increase in prevalence regardless of perinatal time period.^25^ This prevalence increase was only observed in Asian countries particularly in China, Japan and Israel. Notably, this paper reported no significant difference in prevalence changes between developed and developing countries.

In the Philippines, no policy currently supports routine mental health screening and support among perinatal populations, despite its successful implementation of maternal health programmes.^26 27^ This calls for the public health system in the Philippines to reinforce the implementation of its Mental Health Act^28^ to improve the provision of basic and specialised services for pregnant and postpartum women. Our research provides empirical support for the WHO’s recommendations on perinatal mental health integration with maternal and child health services.^29^ The consistently high prevalence of depression across the perinatal period implies a universal approach in early detection, low-intensity interventions and referral to specialised care. An extended surveillance beyond the conventional 12-month postpartum period would also benefit mothers who delayed seeking care.

We found no statistically significant differences in depressive symptoms and prevalence across pregnancy trimesters or months since childbirth. Although perinatal depressive symptoms are generally expected to improve over time, recent evidence indicates that the likelihood of experiencing persistently high or worsening symptoms over the 36 months postpartum is comparable to the likelihood of following an improving mental health trajectory.^14 15^ A cross-country cohort investigation of 11 563 women in the UK, Canada and Singapore showed consistent clinical symptom levels of depression from pregnancy up to 24 months postpartum.^16^ A systematic review of 21 studies from six LMICs and 15 HICs also concluded that maternal depression remained prevalent beyond the first year postpartum, particularly in marginalised groups.^17^ Although depressive symptoms beyond 12 months postpartum may be influenced by non-perinatal factors, persistent depressive symptoms may also be attributed to poor help-seeking behaviour and the limited availability of perinatal mental health support and services for mothers from pregnancy through to the postpartum period and beyond. As such, understanding the broader course of maternal depression is essential for identifying key timepoints for effective intervention and support.

Differences in prevalence were found by age, relationship status and adequacy of prenatal visits. High prevalence among young postpartum mothers may stem from unintended pregnancy, stigma, social isolation and a lack of social support.^30^,^32^ Increased risk for postpartum depression among single mothers was also found in a recent cross-country study in Egypt, Syria, Yemen, Ghana, India and Iraq (AOR=7.09).^33^ Conservative cultural norms and extreme financial pressure of solo parenting may lead to emotional distress and social conflict. These findings underscore the importance of integrating mental health screening and support into maternal and reproductive health services. Providing psychosocial care, parenting support and financial assistance is essential to support the well-being of socially disadvantaged mothers and their children. Furthermore, robust epidemiological data from LMICs are vital to broaden the reach of existing policies and programmes,^34^ extending support beyond only the most high-risk populations to include those at the primary care level. Future research should also identify solutions to address cultural and structural barriers, informing perinatal mental health promotion and prevention strategies.

### Limitations

Our study has a number of limitations. First, we used a non-probability sample of pregnant and postpartum women. This is a common approach in cross-sectional surveys among perinatal populations in LMICs^3^ due to the unavailability of a national sampling frame and short recruitment window, particularly with pregnant women. The gradual reduction in social distancing restrictions in the Philippines at the tail end of the pandemic constrained our access to facilities and communities for population-based recruitment. While our sample is comparable to the Philippines’ perinatal national statistics (online supplemental table S5),^35 36^ results from the survey should be interpreted with caution as our sample is not country-representative. Despite the limited external validity of our research, maternal surveys that used non-probability sampling do not typically produce biased estimates when compared with surveys with population-based samples.^25 37^ Second, it could be argued that online-based recruitment introduced sampling bias, potentially resulting in a skewed sample of mothers with existing mental health problems. However, we did not observe significant differences in item-level responses on the EPDS between those who were recruited online and face-to-face. Although we did not collect comprehensive data on formal psychiatric diagnoses, we asked participants about their use of mental health services and medications. Only two postpartum women reported receiving psychological therapy or counselling, and four reported current use of psychiatric medications. These findings suggest a low likelihood of sampling bias related to mental health status. Due to the extremely small cell sizes, these variables were excluded from the analysis. Finally, EPDS is not a diagnostic instrument and is unable to assess functional impairment related to perinatal depressive symptoms. Although EPDS is considered a gold standard in public health screening, data on its sensitivity and specificity for MDD in LMICs remain insufficient.^38^

### Implications

An alarmingly high prevalence of perinatal depressive symptoms was observed among Filipino mothers, indicating a substantial burden of depression across the antenatal and postpartum periods. The prevalence remained consistent throughout pregnancy and beyond 24 months following childbirth, with elevated risk of postpartum depression among younger women and single mothers. These findings call for integrated public health approaches that incorporate routine perinatal mental health promotion and screening within primary care settings, alongside the timely provision of psychosocial interventions and welfare support for socially disadvantaged populations. Our study offers foundational evidence to guide local and regional research agenda aimed at uncovering the true burden of poor perinatal mental health and identifying evidence-based strategies to enhance the health and well-being of mothers and their children, particularly in low-resource settings.

## Supplementary material

10.1136/bmjopen-2025-109079 online supplemental file 1

## Data Availability

Data are available upon reasonable request.
