# Supplementary material for: Epidemiological investigation of perinatal depression among pregnant and postpartum women: findings from a cross-sectional survey in the Philippines
Source: BMJ Open. 2026 Feb 18;16(2):e109079. doi: 10.1136/bmjopen-2025-109079 (PMC12918685; doi:10.1136/bmjopen-2025-109079)
Supplement: online supplemental file 1 [file bmjopen-16-2-s001.docx]

**Contents**

[Table S1. Distribution of non-response 2](#_Toc218605867)

[Table S2. Full results from multivariable regression analyses among pregnant women 3](#_Toc218605868)

[Table S3. Full results from multivariable regression analyses among postpartum women 4](#_Toc218605869)

[Table S4. Mean EPDS score and prevalence of postpartum depression among women who gave birth within 12 months prior to the survey (N=129) 5](#_Toc218605870)

[Table S5. Comparison between the survey sample and perinatal national statistics in the Philippines, 20 years old or older 6](#_Toc218605871)

[Figure S1. Participant selection flowchart 7](#_Toc218605872)

# Table S1. Distribution of non-response

|  | **Pregnant women (N=356)**  **n** | **Postpartum women (N=500)**  **n** |
| --- | --- | --- |
| **Socio-demographics** |  |  |
| **Current age** | 0 | 0 |
| **Highest educational attainment** | 27 | 13 |
| **Current relationship status** | 14 | 13 |
| **Urbanicity** | 5 | 8 |
| **Obstetric profile** |  |  |
| **First pregnancy status** | 14 | 11 |
| **Main health facility for pregnancy care** | 6 | 6 |
| **Adequate prenatal visits attended** | 10 | 29 |
| **Gestational age** | 3 | 19 |
| **Number of months since birth** | Not applicable | 0 |
| **EPDS items** | 0 | 0 |

# Table S2. Full results from multivariable regression analyses among pregnant women

|  | **Mean (SD)** | ***b* (95% CI)** | **a*b* (95% CI)*** | **% (n)** | **RR (95%CI)** | **aRR (95%CI)**** |
| --- | --- | --- | --- | --- | --- | --- |
| **Current age (in years)** |  |  |  |  |  |  |
| 18-24 | 14.9 (5.4) | 0.32 (-1.09 - 1.73) | 0.22 (-1.47 - 1.91) | 74.4% (67) | 1.10 (0.95 - 1.29) | 1.11 (0.93 - 1.33) |
| 25-34 | 14.6 (5.8) | Ref | Ref | 67.5% (137) | Ref | Ref |
| 35 or older | 12.9 (5.6) | -1.72 (-3.33 - -0.12) | -1.42 (-3.2 - 0.36) | 66.7% (42) | 0.99 (0.81 - 1.21) | 1.03 (0.84 - 1.27) |
| **Highest educational attainment** |  |  |  |  |  |  |
| Secondary or lower | 15.1 (5.2) | Ref | Ref | 75.9% (120) | Ref | Ref |
| University or higher | 13.8 (6.0) | 1.24 (0.02 - 2.46) | 1.08 (-0.32 - 2.47) | 64.9% (111) | 1.17 (1.02 - 1.35) | 1.07 (0.92 - 1.25) |
| **Current relationship status** |  |  |  |  |  |  |
| Married and living with spouse | 13.6 (5.0) | Ref | Ref | 66.5% (107) | Ref | Ref |
| Not married but living with a partner | 14.9 (6.1) | 1.26 (-0.02 - 2.54) | 1.02 (-0.37 - 2.40) | 71.3% (102) | 1.07 (0.92 - 1.25) | 1.03 (0.88 - 1.20) |
| In a relationship but living separately | 14.1 (6.6) | 0.47 (-2.16 - 3.11) | 0.25 (-2.63 - 3.13) | 65.0% (13) | 0.98 (0.70 - 1.37) | 0.91 (0.62 - 1.33) |
| Married but not living with spouse | 15.5 (3.5) | 1.83 (-1.63 - 5.29) | 1.21 (-2.32 - 4.74) | 81.8% (9) | 1.23 (0.91 - 1.66) | 1.14 (0.88 - 1.48) |
| Single | 14.8 (10.5) | 1.21 (-3.41 - 5.82) | -0.31 (-5.42 - 4.79) | 66.7% (4) | 1.00 (0.56 - 1.79) | 0.85 (0.45 - 1.60) |
| Other | NR | NR | NR | NR | NR | NR |
| **Urbanicity** |  |  |  |  |  |  |
| Rural | 14.7 (6.3) | Ref | Ref | 70.5% (62) | Ref | Ref |
| Urban | 14.2 (5.5) | -0.48 (-1.86 - 0.90) | -0.02 (-1.55 - 1.51) | 68.1% (179) | 0.97 (0.82 - 1.13) | 0.96 (0.81 - 1.13) |
| **First pregnancy** |  |  |  |  |  |  |
| Yes | 13.7 (5.8) | -0.91 (-2.21 - 0.39) | -1.11 (-2.62 - 0.39) | 64.2% (70) | 0.91 (0.77 - 1.07) | 0.89 (0.74 - 1.06) |
| No | 14.6 (5.6) | Ref | Ref | 70.8% (165) | Ref | Ref |
| **Main health facility for pregnancy care** |  |  |  |  |  |  |
| Government clinic | 14.3 (6.2) | Ref | Ref | 66.4% (71) | Ref | Ref |
| Government hospital | 14.3 (5.2) | 0.03 (-1.53 - 1.58) | -0.20 (-1.90 - 1.49) | 72.1% (75) | 1.09 (0.91 - 1.30) | 1.09 (0.90 - 1.32) |
| Private (“lying-in”) clinic | 14.2 (5.5) | -0.04 (-1.65 - 1.56) | 0.22 (-1.50 - 1.95) | 68.5% (63) | 1.03 (0.85 - 1.25) | 1.08 (0.89 - 1.32) |
| Private hospital | 14.7 (6.4) | 0.48 (-1.58 - 2.53) | 0.87 (-1.39 - 3.13) | 69.0% (29) | 1.04 (0.82 - 1.33) | 1.03 (0.78 - 1.35) |
| Other | NR | NR | NR | NR | NR | NR |
| **Adequate prenatal visits attended** |  |  |  |  |  |  |
| Yes | 13.9 (5.7) | **-1.80 (-3.17 - -0.45)** | **-1.59 (-3.13 - -0.05)** | 65.4% (168) | **0.80 (0.71 - 0.92)** | 0.88 (0.75 - 1.03) |
| No | 15.7 (5.4) | Ref | Ref | 80.9% (72) | Ref | Ref |
| **Current gestational age** | - | 0.02 (-0.03 - 0.08) | 0.02 (-0.04 - 0.09) | - | 1.00 (0.998 - 1.01) | 1.00 (0.997 - 1.01) |

Notes: SD, standard deviation; %, prevalence based on ≥13 cut-off score; 95% CI, 95% Confidence Interval; NR, Estimates not reported due to denominator ≤5

*Adjusted regression coefficient (a*b*)from multivariable linear regression, adjusted for highest educational attainment, urbanicity, first pregnancy status, type of health facility during pregnancy, adequacy of prenatal visits, and gestational age

**Adjusted risk ratio (aRR) from multivariable negative binomial regression, adjusted for highest educational attainment, urbanicity, first pregnancy status, type of health facility during pregnancy, adequacy of prenatal visits, and gestational age

# Table S3. Full results from multivariable regression analyses among postpartum women

|  | **Mean (SD)** | ***b* (95% CI)** | **a*b* (95% CI)*** | **% (n)** | **RR (95%CI)** | **aRR (95%CI)**** |
| --- | --- | --- | --- | --- | --- | --- |
| **Current age (in years)** |  |  |  |  |  |  |
| 18-24 | 15.8 (5.5) | 1.32 (-0.14 - 2.77) | **1.96 (0.30 - 3.61)** | 72.9% (62) | 1.16 (0.99 - 1.36) | **1.23 (1.03 - 1.47)** |
| 25-34 | 14.4 (6.2) | Ref | Ref | 62.9% (193) | Ref | Ref |
| 35 or older | 12.0 (6.1) | **-2.44 (-3.77 - -1.11)** | **-2.24 (-3.65 - -0.82)** | 50.9% (55) | 0.81 (0.66 - 0.99) | 0.86 (0.69 - 1.06) |
| **Highest educational attainment** |  |  |  |  |  |  |
| Secondary or lower | 14.5 (5.8) | Ref | Ref | 66.2% (180) | Ref | Ref |
| University or higher | 13.7 (6.6) | 0.81 (-0.29 - 1.91) | 0.91 (-0.27 - 2.09) | 58.1% (125) | 1.14 (0.99 - 1.31) | 1.13 (0.97 - 1.31) |
| **Current relationship status** |  |  |  |  |  |  |
| Married and living with spouse | 13.4 (6.1) | Ref | Ref | 58.6% (143) | Ref | Ref |
| Not married but living with a partner | 15.0 (6.0) | **1.61 (0.46 - 2.76)** | **1.53 (0.33 - 2.73)** | 66.7% (130) | 1.14 (0.98 - 1.31) | 1.12 (0.96 - 1.31) |
| In a relationship but living separately | 16.3 (6.1) | 2.94 (-1.13 - 7.01) | 3.08 (-1.19 - 7.35) | 77.8% (7) | 1.33 (0.92 - 1.91) | 1.41 (0.99 - 2.03) |
| Married but not living with spouse | 13.3 (5.9) | -0.06 (-3.25 - 3.13) | 0.62 (-2.66 - 3.89) | 60.0% (9) | 1.02 (0.67 - 1.57) | 1.07 (0.67 - 1.70) |
| Single | 16.3 (7.9) | 2.92 (-0.17 - 6.01) | **3.46 (0.22 - 6.71)** | 75.0% (12) | 1.28 (0.95 - 1.73) | **1.42 (1.07 - 1.90)** |
| Other | 16.3 (5.1) | 2.86 (-1.45 - 7.16) | 1.08 (-3.81 - 5.97) | 75.0% (6) | 1.28 (0.85 - 1.94) | 1.06 (0.64 - 1.74) |
| **Urbanicity** |  |  |  |  |  |  |
| Rural | 14.9 (5.7) | Ref | Ref | 64.3% (63) | Ref | Ref |
| Urban | 13.9 (6.2) | -1.05 (-2.41 - 0.32) | -1.25 (-2.69 - 0.19) | 60.9% (240) | 0.95 (0.80 - 1.12) | 0.91 (0.76 - 1.08) |
| **First pregnancy** |  |  |  |  |  |  |
| Yes | 14.7 (6.2) | 0.91 (-0.28 - 2.10) | 0.13 (-1.26 - 1.52) | 67.1% (100) | 1.13 (0.98 - 1.30) | 1.09 (0.92 - 1.29) |
| No | 13.8 (6.2) | Ref | Ref | 59.4% (202) | Ref | Ref |
| **Main health facility for pregnancy care** |  |  |  |  |  |  |
| Government clinic | 14.4 (6.0) | Ref | Ref | 65.6% (105) | Ref | Ref |
| Government hospital | 14.3 (6.1) | -0.10 (-1.50 - 1.30) | -0.17 (-1.62 - 1.28) | 60.8% (87) | 0.93 (0.78 - 1.10) | 0.90 (0.75 - 1.08) |
| Private (“lying-in”) clinic | 13.8 (6.2) | -0.58 (-2.03 - 0.87) | -0.79 (-2.34 - 0.76) | 59.5% (75) | 0.91 (0.76 - 1.09) | 0.88 (0.71 - 1.07) |
| Private hospital | 13.3 (6.9) | -1.09 (-2.93 - 0.76) | 0.04 (-1.88 - 1.96) | 56.7% (34) | 0.86 (0.67 - 1.11) | 0.94 (0.73 - 1.21) |
| Other | NR | NR | NR | NR | NR | NR |
| **Adequate prenatal visits attended** |  |  |  |  |  |  |
| Yes | 13.2 (6.3) | **-1.78 (-2.89 - -0.67)** | **-1.30 (-2.48 - -0.12)** | 52.0% (148) | **0.79 (0.69 - 0.91)** | **0.83 (0.72 - 0.97)** |
| No | 15.0 (5.8) | Ref | Ref | 69.3% (140) | Ref | Ref |
| **Gestational age** |  | -0.02 (-0.32 - 0.28) | -0.04 (-0.34 - 0.26) |  | 1.00 (0.96 - 1.04) | 0.99 (0.95 - 1.03) |
| **Number of months since birth** |  | 0.02 (-0.02 - 0.05) | 0.02 (-0.02 - 0.05) |  | 1.00 (0.998 - 1.01) | 1.00 (0.997 - 1.01) |

Notes: SD, standard deviation; %, prevalence based on ≥13 cut-off score; 95% CI, 95% Confidence Interval; NR, Estimates not reported due to denominator ≤5

*Adjusted regression coefficient (a*b*) from multivariable linear regression, adjusted for highest educational attainment, urbanicity, first pregnancy status, type of health facility during pregnancy, adequacy of prenatal visits, gestational age since birth of reference pregnancy, number of months since birth of reference pregnancy

**Adjusted risk ratio (aRR) from multivariable negative binomial regression, adjusted for highest educational attainment, urbanicity, first pregnancy status, type of health facility during pregnancy, adequacy of prenatal visits, gestational age since birth of reference pregnancy, number of months since birth of reference pregnancy

# Table S4. Mean EPDS score and prevalence of postpartum depression among women who gave birth within 12 months prior to the survey (N=129)

| **Time since delivery** | **N** | **Mean EPDS score (95% CI)** | **Prevalence (95% CI)** |
| --- | --- | --- | --- |
| 0-3 months | 48 | 13.3 (11.6 - 14.9) | 54.2 (40.0 - 67.7) |
| 4-6 months | 30 | 12.7 (10.3 - 15.0) | 50.0 (32.7 - 67.3) |
| 7-9 months | 31 | 15.4 (13.6 - 17.2) | 71.0 (52.8 - 84.2) |
| 10-12 months | 20 | 15.9 (13.9 - 17.9) | 75.0 (51.9 - 89.3) |

# Table S5. Comparison between the survey sample and perinatal national statistics in the Philippines, 20 years old or older

|  | **Survey (Postpartum mothers)** | **Based on the number of registered livebirths in the Philippines, 2023^[[1]](#footnote-1)^** |
| --- | --- | --- |
| **Age (in years)** |  |  |
| 20-24 | 25.9% | 24.6% |
| 25-34 | 54.8% | 56.7% |
| 35+ | 19.3% | 18.7% |
| **Marital status** |  |  |
| Married | 53.2% | 45.4% |
| Not married | 46.9% | 54.6% |
| **Location** |  |  |
| Luzon | 69.2% | 46.5% |
| National Capital Region | 22.9% | 11.7% |
| Visayas | 5.4% | 18.3% |
| Mindanao | 2.5% | 23.6% |
| **Highest educational attainment*** |  |  |
| Secondary or lower | 54.8% | 63.8%^+^ |
| University or higher | 45.2% | 36.2%^+^ |
| **First pregnancy** |  |  |
| Yes | 29.1% | 29.0% *(1^st^ birth order)* |
| No | 70.9% | 71.0% *(2^nd^ and over)* |
| **Number of prenatal visits attended** |  |  |
| <4 | 37.6% | 16.3%^+^ |
| 4+ | 62.4% | 83.7%^+^ |

*Include <20 years old; ^+^Based on the 2022 Philippines National Demographic and Health Survey^[[2]](#footnote-2)^

# Figure S1. Participant selection flowchart

**2924 reached and screened**

- 163 via face-to-face from 7 maternal care facilities
- 461 organically via Facebook (i.e., snowballed)
- 2300 via Facebook ads

**Pregnant women**

365 eligible

59

via face-to-face

306

via online, provided with the survey link

**Postpartum women**

572 eligible

91

via face-to-face

481

via online, provided with the survey link

59

301

**Started the survey**

**360 in total**

**Response rate = 98.6%**

91

428

**519 in total**

**Response rate = 90.7%**

59

297

(288+ 9*)

**Completed the survey**

Final sample

**356 pregnant women**

91

409

(401 + 8*)

Final sample

**500 postpartum women**

**1987 were not eligible pregnant and postpartum women**

- 692 screened as eligible for other surveys in COCOON
- 73 were not further contacted due to potentially fraudulent Facebook accounts and mismatched pregnancy information provided during the initial chat/contact
- 1222 had never been pregnant or given birth before 30 January 2020

**Partially completed the survey, at least the psychosocial wellbeing module where EPDS sits*

**58 online participants were non-contactable after the survey link was provided**

- 5 pregnant
- 53 postpartum

1. Philippine Statistics Authority. Registered live births in the Philippines, 2023. 20 December 2024 ed. Quezon City, Philippines: Philippine Statistics Authority, 2024. [↑](#footnote-ref-1)
2. Philippine Statistics Authority and ICF. 2022 Philippine National Demographic and Health Survey (NDHS): Final report. Quezon City, Philippines and Rockville, Maryland, USA: Philippine Statistics Authority and ICF, 2023. [↑](#footnote-ref-2)
