# Supplementary material for: Epidemiological investigation of perinatal depression among pregnant and postpartum women: findings from a cross-sectional survey in the Philippines
Source: BMJ Open. 2026 Feb 18;16(2):e109079. doi: 10.1136/bmjopen-2025-109079 (PMC12918685; doi:10.1136/bmjopen-2025-109079)
Supplement: Supplementary file 2 [file bmjopen-2025-109079.reviewer_comments.pdf]

PEER REVIEW HISTORY

BMJ Open publishes all reviews undertaken for accepted manuscripts. Reviewers are asked to complete a checklist review form and are provided with free text boxes to elaborate on their assessment. These free text comments are reproduced below.

ARTICLE DETAILS

Title (Provisional)

Epidemiological investigation of perinatal depression among pregnant and postpartum women: Findings from a cross-sectional survey in the Philippines

Authors

Filoteo, Joycelyn Abiog; Maravilla, Joemer Calderon; Mamaat, Jose Edwardo; Flores, Arbie Diane; Jumamil, Ana Nelia; Cardenas, Reinalyn Lim; Quijencio, Jr., Wilfredo; Bayani, Mary Ann; Santos, Jr., Nemencio; Acena, Joyce Lisa; Alfonso, Anna Liza; Rivera, Mayla; Guarino, Rebecca; Garcia, Rachelle; Flenady, Vicki; Boyle , Frances M; Loughnan, Siobhan A; Taragua, Alma

---

VERSION 1 - REVIEW

**Reviewer**	**1**
**Name**	**Matsumura, Kenta**
**Affiliation**	**University of Toyama, Public Health**
**Date**	**22-Sep-2025**
**COI**	**None**

This cross-sectional study from the Philippines clarifies the prevalence of postpartum depression during and after the COVID-19 pandemic and its associated factors. While the research background itself is understandable, the current methodology contains unclear points, and the description is insufficient. Significant revisions are necessary as there is considerable room for improvement regarding scientific validity. Comments follow.

More detailed information is needed on how participants were recruited for this study. Crucially, what was the timeframe of the survey? This should be clearly stated. While Ref 8 is cited, the complete lack of discussion on its specific content is problematic. If community-based recruitment is one of this study's strengths, a minimum explanation of how this was ensured is necessary. Facebook is mentioned, but if recruitment targeted only Facebook users, that would be internet-based, not community-based. Internet-based recruitment may be more likely to include participants who are relatively young, isolated from real-world social networks, or have other characteristics associated with higher postpartum depression prevalence. How is this considered? Furthermore, if recruitment methods included both web-based and face-to-face approaches, differences due to recruitment method must be carefully examined. If the study spanned multiple years, the impact of survey timing must also be considered. Adding this information to Table 1 should be considered. In any case, the

authors must provide more detailed explanations, use more appropriate terminology, and conduct appropriate additional analyses.

Create a participant selection flowchart. The attached STROBE checklist item 13(c) states it is included in the supplement, but it is actually missing. The same applies to item 13(b). The authors should verify thoroughly before submission and ensure all items are correctly marked on the checklist.

Table S1 is unclear. Are 356 and 500 not non-response rates but rather the number of responders? The authors should rewrite to clarify.

Figures S1 and S2 do not match the references in the main text. Could the wrong figures have been included?

The abbreviation "ACoef" is not standard. Typically, regression analysis coefficients are referred to as β coefficients. The authors should use the term "β" and reconsider the abbreviation.

Include crude estimates (crude odds ratios and crude β coefficients). Similar to the previous point, STROBE checklist 16(a) is marked as reported, but it is not actually reported. If the authors notice during the pre-submission check that something is not reported, they should properly revise the manuscript before entering the page numbers into the STROBE checklist.

The handling of the EPDS cutoff is unusual. In the literature "Using the Edinburgh Postnatal Depression Scale (EPDS): Translated into languages other than English" it is stated that the cutoff for the Filipino catalog language version is 9/10.

https://www.mcpapformoms.org/Docs/Edinburgh%20Depression%20Scale%20Translated%20Government%20of%20Western%20Australia%20Department%20of%20Health.pdf

However, this value is from a 2006 publication and is listed as a recommended value without a validity study. If a subsequent paper on the Philippine version's cutoff value (a study clarifying sensitivity, specificity, and cutoff value against the EPDS gold standard) has been published, the authors should use that value. Alternatively, if based on Ref 13, the cutoff value should be 11. Even when using this value, the rationale for its adoption (e.g., lack of validation studies for the catalog language version) should be explicitly stated in the methods. Additionally, analyses using other strong cutoff values that were not adopted should be performed as sensitivity analyses. Ideally, a citation of the validation study for the Philippine version EPDS cutoff value mentioned earlier is required.

If no validation study for the EPDS cutoff value exists for the target population, it would be preferable to conduct the main analysis using continuous values. Treating the data as symptoms rather than prevalence seems likely to cause fewer problems.

When prevalence is very high, as in this study, calculating odds ratios is not ideal. The authors should use risk ratios instead of odds ratios.

Setting aside the issue of the EPDS cutoff value, the high prevalence rate in this study appears to stand out even compared to other Philippine studies. However, the explanation for this reason lacks persuasiveness. Rather than the issue of timing (within 12 months postpartum) mentioned in the second paragraph of the discussion, the impact of the pandemic and the sampling method seem to be stronger factors. Several studies have

reported increased postpartum depression prevalence during the pandemic; the authors should cite and discuss these.

Provide more detail in the Methods section on how trend analysis was performed.

The Abstract lists both point estimates for ACoef and AOR, but the critical information is the 95% confidence intervals. The authors should add these.

Reference 6: The author name and page number are incorrect. The authors should thoroughly check all reference information for similar errors.

There is no need to duplicate the same content as shown in Fig 1 and STable 3; typically, a table alone is sufficient.

**Reviewer**	**2**
**Name**	**Dama, Manish**
**Affiliation**	**Trinity College Dublin School of Medicine**
**Date**	**30-Sep-2025**
**COI**	**None**

I would like to thank the authors for quantifying the prevalence of self-reported perinatal depression in the Philippines towards the end of the COVID-19 pandemic. The findings of this study are very important for public health policy in the Philippines as it reveals alarmingly high rates of self-reported depressive symptoms among pregnant (59.3%) and postpartum (56.4%) persons, respectively. Despite the importance of their findings, the manuscript would benefit from the following revisions prior to being considered for publication.

1) The authors need to restrict their postpartum sample to those who were assessed up to 12 months postpartum. Perinatal depression is typically defined as the onset of a major depressive episode during any point in pregnancy and up to 12 months postpartum. It is possible that some of the participants who are experiencing self-reported depression after 12 months postpartum may have developed depressive symptoms due to non-perinatal reasons.

In addition to this restriction, it would be useful to report the number of participants in the following subgroups: A) 0-3 months, B) 4-6 months, C) 7-9 months, and D) 10-12 months. In addition to reporting this, the authors may consider using a histogram for its visualization as well.

Furthermore, reporting their mean Edinburgh Postnatal Depression Scale (EPDS) score and the prevalence of self-reported depression in these postpartum subgroups would be useful as well. With these results, the authors can more appropriately compare their findings from the 0-3 month (0-12 weeks) subgroup to the results seen in the two studies that examined postpartum depression rates in the Philippines (the study samples in these two studies included postpartum persons up to 6 and 8 weeks, respectively).

2) It would also be useful to the reader to report the number of participants in each trimester for the pregnancy sample. In addition to reporting this, the authors may consider using a histogram for its visualization as well.

3) The authors should provide a flowchart describing how many individuals were approached for the study, how many agreed, how many were excluded from the study based on their eligibility criteria, and how many were included in the study sample. The authors should then statistically compare the study sample to those who were excluded based on the eligibility criteria in regards to sociodemographic data. This will determine if their study sample is representative of the study population.

4) If the authors have access to data on the State-Trait Anxiety Inventory Scale, Perceived Stress Scale-4, and De Jon Gierveld Loneliness Scale, they should consider reporting these scores as well in their study. The COCOON Global Collaboration protocol (which the current study is apart of) used these scales in addition to the EPDS. The level of anxiety, stress, and social loneliness among participants may help explain the alarmingly high rates of self-reported depression in pregnancy and the postpartum within this study. If present, the authors should report these scores and statistically compare them among those with self-reported depression and those without self-reported depression.

5) The authors should consider reporting that a limitation of their study is the lack of data on psychiatric (e.g., personal history of mental disorders) and obstetric (e.g., gestational complications) factors in their study. There could be differences in these factors among those with self-reported depression and those without self-reported depression.

6) Lines 15 to 19 on page 14 in the Discussion needs to be removed as the authors did not examine this in their study based on their Methodology and Results sections.

7) Minor revisions: A) Please report the sample size for the whole study sample as well as the pregnancy and postpartum subsamples in the Findings section of the Abstract; B) Please report the sample size for reference 7 in lines 21-22 on page 14 of the Discussion; C) Please report the prevalence rate reported for reference 15 in lines 36-40 on page 14 of the Discussion.

**Reviewer**	**3**
**Name**	**Papadopoulos, Fotios**
**Affiliation**	**Uppsala Univ, Department of Medical Sciences, Psychiatry**
**Date**	**06-Oct-2025**
**COI**	**None**

General comment

I would like to congratulate the authors on conducting a much-needed research project on the prevalence of perinatal depression in the Philippines. The article is well written, with clearly described methods and results. I only have a few minor comments that I believe could help improve the clarity of the methodology and better contextualize the findings.

Background / Discussion

- Consider including a broader discussion of mental health conditions among Filipino women, either in the background or discussion section. This would provide important context, especially given the alarmingly high prevalence rates reported during pregnancy and postpartum.

Methods

- The description of women with miscarriage, stillbirth, or neonatal death appears inconsistent. Were these women excluded from the analysis? If so, why is it stated in the discussion that their small number would not have substantially affected the results?
- Please indicate how many maternal care facilities were involved in recruitment, and from which geographical regions.
- The manuscript states that "an internal consistency" was calculated for the EPDS during pregnancy and postpartum, but no details of the statistical method are provided. Please specify the type of reliability coefficient used (e.g., Cronbach's alpha).
- On page 6, lines 33–35, the authors describe verifying participants' names, gestational age, and delivery date. How was this verification conducted? Which databases or administrative records were accessed?
- A flowchart would greatly improve transparency. On page 8, line 17, it is stated that "all participants responded to all EPDS items," whereas line 46 indicates that only responses with at least one completed EPDS were included. A flow diagram would clarify exclusions due to missing EPDS data.
- Has the EPDS been validated in the Philippines? If not, are there validation studies or cutoff recommendations from culturally comparable populations? This is particularly relevant given the high prevalence estimates reported.
- Please clarify how recruitment via social media was conducted. Is it possible that women experiencing mental health difficulties were more likely to respond to such outreach, which could partially explain the high prevalence?

Results

- The finding that "most pregnant women had previous pregnancies (68.1%), while postpartum women were primarily experiencing their first pregnancy (69.5%)" appears contradictory. This raises concerns about how parity was assessed. It is conceivable that postpartum women excluded their current pregnancy from the count—if so, please clarify the wording of this question.
- In Table 1, some percentage values appear to be missing. Please ensure all percentage values are presented unless truly zero.

Discussion

- Please elaborate on the generalizability of the findings. Are there national statistics on perinatal characteristics such as maternal age, gestational age at delivery, or relationship status? If so, including or citing these would strengthen the paper by demonstrating how representative the study population is.
- Throughout the manuscript, consider replacing the term *depression* with *depressive symptoms*, as the EPDS is a screening tool rather than a diagnostic

instrument.

- The potential influence of the COVID-19 pandemic on the results should be discussed more explicitly. Could elevated symptom levels reflect pandemic-related stressors?
- Expand the comparison with previous studies from the Philippines. What factors might explain the discrepancy in prevalence—differences in setting, timing (pre- vs post-pandemic), population characteristics, or methodology?

Overall, this is an important and timely contribution. Addressing the points above would further strengthen the manuscript.

VERSION 1 - AUTHOR RESPONSE

Reviewer 1

**Comment**	**Response**
This cross-sectional study from the Philippines clarifies the prevalence of postpartum depression during and after the COVID-19 pandemic and its associated factors. While the research background itself is understandable, the current methodology contains unclear points, and the description is insufficient. Significant revisions are necessary as there is considerable room for improvement regarding scientific validity. Comments follow.	We thank the Reviewer for this feedback. We have outlined our revisions to the manuscript in the following responses.
More detailed information is needed on how participants were recruited for this study. Crucially, what was the timeframe of the survey? This should be clearly stated. While Ref 8 is cited, the complete lack of discussion on its specific content is problematic. ... If the study spanned multiple years, the impact of survey timing must also be considered.	We mentioned in the Methods section (page 6) the contents of the global protocol of the COCOON study, which was published in BMJ Open.<br><br>“A cross-sectional survey was developed in partnership with the COCOON Global Collaboration, coordinated by Australia’s National Health and Medical Research Council (NHMRC) Centre of Research Excellence in Stillbirth (Stillbirth CRE) in Brisbane, Australia. COCOON’s published global protocol discussed the countries involved in the survey, the overarching survey methodology and measures, and the anticipated survey administration period.[9] ”

Comment	Response
	The survey was conducted over a six-month period, from November 2023 to May 2024. This is further highlighted in the Methods section (pages 6-7).<br><br>"**Survey development and procedure**<br>The survey was initially developed at the beginning of the COVID-19 pandemic as part of a multi-country collaboration to examine parent psychosocial well-being and experiences of maternity, neonatal, and perinatal bereavement care. In the Philippines, the survey was further adapted in the local setting in 2022-2023. ... The data collected lasted for six months, between November 2024 to May 2024."<br><br>In reference to the survey administration period mentioned in COCOON's global protocol (Loughnan et al., 2022), the actual implementation of the survey in the Philippines was delayed due to institutional administrative issues, ethics review, and translation of the questionnaires into Filipino/Tagalog.
If community-based recruitment is one of this study's strengths, a minimum explanation of how this was ensured is necessary. Facebook is mentioned, but if recruitment targeted only Facebook users, that would be internet-based, not community-based. Internet-based recruitment may be more likely to include participants who are relatively young, isolated from real-world social networks, or have other characteristics associated with higher postpartum depression prevalence. How is this considered? Furthermore, if recruitment methods included both web-based and face-to-face approaches, differences due to recruitment method must be carefully examined. Adding this information to Table 1 should be considered. In any case, the authors must provide more detailed explanations, use more	The survey applied both online-based and community-based recruitment strategies. Facebook was selected as the sole online platform for recruiting participants. Knowing that ~100% of 18-year-olds and above in the Philippines use Facebook (Kemp, 2025), this platform enabled effective recruitment of our target participants while minimising the impact of stigma on participation. We have added this explanation to the Methods section (page 6).<br><br>"Facebook is considered the most used social media platform by Filipinos 18 years or older and Facebook ads have been documented to reach 93.1% of users in the Philippines.[10]..."<br><br>The main intention of Table 1 is to describe the sample in terms of various covariates considered in the study. The participant distribution, in terms of recruitment mode, is now presented as part of our participant selection flowchart (Figure S1) and also discussed in text in the Findings section (page 9).<br><br>"A total of 856 women who participated in the survey were included in the final sample, which comprised 356 pregnant women and 500 postpartum women, with 98.6% and 90.7% response rates (see Figure S1). The majority of pregnant (n = 297, 83.4%) and postpartum women (n = 409, 81.8%) in the final sample were recruited online, noting that the mode of

Comment	Response
appropriate terminology, and conduct appropriate additional analyses.	recruitment was not statistically different between participant types (X2=0.38, p=0.537).”<br><br>For transparency, we examined the differences in responses for each EPDS item between participants recruited online and those recruited face-to-face, by participant type. We found no statistical difference in response patterns between modes of recruitment except for item 10 (p=0.034) among pregnant women, and items 2 (p=0.046), item 8 (p=0.036), and item 10 (p=0.017) among postpartum women. Higher reports of self-harm behaviour (item 10) among online participants (pregnant: 33.7% vs 23.7%; postpartum: 34.4% vs 22.0%) are expected due to the known stigma on this behaviour. As such, we don’t anticipate that the mode of recruitment will confound the study results.
Create a participant selection flowchart. The attached STROBE checklist item 13(c) states it is included in the supplement, but it is actually missing. The same applies to item 13(b). The authors should verify thoroughly before submission and ensure all items are correctly marked on the checklist.	We created the participant selection flowchart for our study, as shown in Figure S1, and included it as part of the supplemental file.
Table S1 is unclear. Are 356 and 500 not non-response rates but rather the number of responders? The authors should rewrite to clarify.	We apologise for the confusion. Table S1 outlines the number of non-responses per variable listed in the table. The N row is intended to help readers calculate the % of non-response per variable using N as the denominator. We removed the N row to avoid confusion.
Figures S1 and S2 do not match the references in the main text. Could the wrong figures have been included?	We have removed these figures to avoid confusion.
The abbreviation “ACoef” is not standard. Typically, regression analysis coefficients are referred to as β coefficients. The authors should use the term “β” and reconsider the abbreviation.	We believe that Betas (β) are usually referred to as standardised coefficients, which isn’t the case in the study. We decided to use “b” to indicate crude regression coefficients and “ab” for adjusted regression coefficients instead.

Comment	Response
Include crude estimates (crude odds ratios and crude β coefficients). Similar to the previous point, STROBE checklist 16(a) is marked as reported, but it is not actually reported. If the authors notice during the pre-submission check that something is not reported, they should properly revise the manuscript before entering the page numbers into the STROBE checklist.	We have now included the crude or unadjusted regression results in Table 2, Table 3, Table S2, and Table S3.
The handling of the EPDS cutoff is unusual. In the literature "Using the Edinburgh Postnatal Depression Scale (EPDS): Translated into languages other than English" it is stated that the cutoff for the Filipino catalog language version is 9/10. https://www.mcpapformoms.org/Docs/Edinburgh%20Depression%20Scale%20Translated%20Government%20of%20Western%20Australia%20Department%20of%20Health.pdf However, this value is from a 2006 publication and is listed as a recommended value without a validity study. If a subsequent paper on the Philippine version's cutoff value (a study clarifying sensitivity, specificity, and cutoff value against the EPDS gold standard) has been published, the authors should use that value. Alternatively, if based on Ref 13, the cutoff value should be 11. Even when using this value, the rationale for its adoption (e.g., lack of validation studies for the catalog language version) should be explicitly stated in the methods. Additionally, analyses using other strong cutoff values	We decided not to use the cut-off from this reference, as this was not based on local validation studies. The most recent diagnostic validation in the Philippines was conducted in 2010 (Balce-Mercado, 2011). This study suggested a cut-off of ≥13 with 84% sensitivity and 85% specificity. We have now used this as our cut-off score for all regression analysis.

Comment	Response
that were not adopted should be performed as sensitivity analyses. Ideally, a citation of the validation study for the Philippine version EPDS cutoff value mentioned earlier is required.<br><br>If no validation study for the EPDS cutoff value exists for the target population, it would be preferable to conduct the main analysis using continuous values. Treating the data as symptoms rather than prevalence seems likely to cause fewer problems.	
When prevalence is very high, as in this study, calculating odds ratios is not ideal. The authors should use risk ratios instead of odds ratios.	We agree with the Reviewer's suggestion. As discussed in the Methods section (page 8), we have analysed the prevalence using negative binomial regression to generate Risk Ratios (RRs).<br><br>"Multivariable linear and negative binomial regression analyses were conducted to examine epidemiological patterns of depressive symptoms, using the EPDS as a continuous score and a binary variable, respectively..."
Setting aside the issue of the EPDS cutoff value, the high prevalence rate in this study appears to stand out even compared to other Philippine studies. However, the explanation for this reason lacks persuasiveness. Rather than the issue of timing (within 12 months postpartum) mentioned in the second paragraph of the discussion, the impact of the pandemic and the sampling method seem to be stronger factors. Several studies have reported increased postpartum depression prevalence during the pandemic; the authors should cite and discuss these.	We thank the Reviewer for the suggestion to strengthen our Discussion. Relevant texts in the Discussion (pages 14-15) have been revised.<br><br>"The differences in prevalence between our study and previous research may be explained by the profound impact of the pandemic on women's mental health as well as the persistent gaps in perinatal mental health services in the Philippines. The Global Burden of Disease study reported a disproportionate rise in major depressive disorder (MDD) prevalence among women compared to men, with the Philippines experiencing the highest increase in MDD prevalence across Southeast Asia.[24] A recent meta-analysis of 22 studies comparing postpartum depression prevalence before and during the pandemic found a significant increase in prevalence regardless of perinatal time period.[25] This prevalence increase was only observed in Asian countries particularly in China, Japan and Israel. Notably, this paper reported no significant difference in prevalence changes between developed and developing countries.<br><br>In the Philippines, no policy currently supports routine mental health screening and support among perinatal populations,

Comment	Response
	despite its successful implementation of maternal health programs.[26 27] This calls for the public health system in the Philippines to reinforce the implementation of its Mental Health Act[28] to improve the provision of basic and specialised services for pregnant and postpartum women. Our research provides empirical support for the World Health Organization's recommendations on perinatal mental health integration with maternal and child health services.[29] The consistently high prevalence of depression across the perinatal period implies a universal approach in early detection, low-intensity interventions, and referral to specialised care. An extended surveillance beyond the conventional 12-month postpartum period would also benefit mothers who delayed seeking care.<br><br>We found no statistically significant differences in depressive symptoms across pregnancy trimesters or months since childbirth, suggesting a persistently elevated risk throughout the perinatal period. Although perinatal depressive symptoms are generally expected to improve over time, recent evidence indicates that the likelihood of experiencing persistently high or worsening symptoms over the 36 months postpartum is comparable to the likelihood of following an improving mental health trajectory.[14 15] A cross-country cohort investigation of 11,563 women in the UK, Canada and Singapore showed consistent clinical symptom levels of depression from pregnancy up to 24 months postpartum.[16] A systematic review of 21 studies from six LMICs and 15 HICs also concluded that maternal depression remained prevalent beyond the first year postpartum, particularly in marginalised groups.[17] Although depressive symptoms beyond 12 months postpartum may be influenced by non-perinatal factors, persistent depressive symptoms may also be attributed to poor help-seeking behaviour and the limited availability of perinatal mental health support and services for mothers from pregnancy through to the postpartum period and beyond. As such, understanding the broader course of maternal depression is essential for identifying key timepoints for effective intervention and support."
Provide more detail in the Methods section on how trend analysis was performed.	Trend analysis was conducted by regressing perinatal time period as an ordinal time variable. Perinatal time period variable was generated using the age of gestation among pregnant women and the months after birth among postpartum women (time variable categories: 1st, 2nd, and 3rd trimesters, 0-12 months, 13-24 months, and 25+ months postpartum). The estimates were further adjusted for demographic

Comment	Response
	characteristics and other pregnancy information. This was further explained in the Methods section (page 8).<br><br>"...Regression analyses were used to examine the trend of depressive symptoms and prevalence by perinatal time period and by timing of birth (among postpartum women). The timing of birth was categorised into two groups: during the pandemic and after the pandemic (from July 2023 onward), reflecting the lifting of the COVID-19 public health emergency status by the Philippine government in July 2023.[21] Analysis by perinatal time period was performed using the combined pregnant and postpartum sample, with a time variable generated based on AOG among pregnant women and months after birth among postpartum women (time variable categories: 1st, 2nd, and 3rd trimesters, 0-12 months, 13-24 months, and 25+ months postpartum). All analyses were adjusted for demographic characteristics and obstetric profile."
The Abstract lists both point estimates for ACoef and AOR, but the critical information is the 95% confidence intervals. The authors should add these.	We have included the 95% Confidence Interval of the regression results presented in the Abstract.<br><br>"**Results:** A total of 856 women participated in the study, comprising 356 pregnant and 500 postpartum women. EPDS scores were, on average, similar between pregnant (14.4) and postpartum women (14.1). Using the locally validated cut-off of ≥13 revealed that 69.1% of pregnant and 62.0% of postpartum women reported clinically significant depressive symptoms. Consistent EPDS scores and prevalence were observed across pregnancy trimesters and within12 months postpartum and beyond. Women who received adequate prenatal care were less likely to experience antenatal (ab=-1.59, 95%CI=-3.13--0.05) and postpartum (ab=-1.30, 95% CI=-2.48--0.12) depressive symptoms. Postpartum EPDS scores and depressive symptom prevalence (EPDS score ≥13) were higher among 18–24-year-olds (ab=1.96, 95% CI=0.30 - 3.61; aRR=1.23, 95% CI=1.03 - 1.47) and single mothers (ab=3.46, 95% CI=0.22 - 6.71; aRR=1.42, 95% CI=1.07 - 1.90), compared to older and married mothers respectively."
Reference 6: The author name and page number are incorrect. The authors should thoroughly check all reference information for similar errors.	All references have been checked for any errors. We confirm that the details in the reference list are all correct.
There is no need to duplicate the same content as shown in Fig 1	Thank you for the suggestion. We decided to retain the table and move it from the supplemental file to the main manuscript file as Table 2.

Comment	Response
and STable 3; typically, a table alone is sufficient.	

Reviewer 2

Comment	Response
I would like to thank the authors for quantifying the prevalence of self-reported perinatal depression in the Philippines towards the end of the COVID-19 pandemic. The findings of this study are very important for public health policy in the Philippines as it reveals alarmingly high rates of self-reported depressive symptoms among pregnant (59.3%) and postpartum (56.4%) persons, respectively. Despite the importance of their findings, the manuscript would benefit from the following revisions prior to being considered for publication.	We thank the Reviewer for this feedback. We have outlined our revisions to the manuscript in the following responses.
1) The authors need to restrict their postpartum sample to those who were assessed up to 12 months postpartum. Perinatal depression is typically defined as the onset of a major depressive episode during any point in pregnancy and up to 12 months postpartum. It is possible that some of the participants who are experiencing self-reported depression after 12 months postpartum may have developed depressive symptoms due to non-perinatal reasons.	We understand that perinatal depression is typically defined as the onset of a major depressive episode during pregnancy and up to 12 months postpartum. However, a growing number of studies have highlighted the importance of considering the prevalence and monitoring depressive symptoms beyond the first postpartum year, as some women continue to experience clinically significant symptoms beyond 12 months. Putnick et al. (2020) highlighted that postpartum depressive symptoms can persist for up to 36 months after childbirth and may follow different trajectories. A cross-country cohort investigation of 11,563 women in the UK, Canada and Singapore also showed consistent clinical symptom levels of depression from pregnancy up to 24 months postpartum (Kee et al., 2023). A systematic review of 21 studies also concluded that maternal depression remained prevalent beyond the first postpartum year, particularly in marginalised groups (Hunter et al., 2024). Implications for both maternal well-being and child development are significant, underlining the importance of an expanded period of measuring the prevalence of postpartum depression. Our study findings support these observations as our study found that the depressive symptoms remain

Comment	Response
	similar across antenatal and postpartum women, within and beyond 12 months postpartum.<br><br>These are included in the Discussion section (page 15).<br><br>"We found no statistically significant differences in depressive symptoms across pregnancy trimesters or months since childbirth, suggesting a persistently elevated risk throughout the perinatal period. Although perinatal depressive symptoms are generally expected to improve over time, recent evidence indicates that the likelihood of experiencing persistently high or worsening symptoms over the 36 months postpartum is comparable to the likelihood of following an improving mental health trajectory.[14 15] A cross-country cohort investigation of 11,563 women in the UK, Canada and Singapore showed consistent clinical symptom levels of depression from pregnancy up to 24 months postpartum.[16] A systematic review of 21 studies from six LMICs and 15 HICs also concluded that maternal depression remained prevalent beyond the first year postpartum, particularly in marginalised groups.[17] Although depressive symptoms beyond 12 months postpartum may be influenced by non-perinatal factors, persistent depressive symptoms may also be attributed to poor help-seeking behaviour and the limited availability of perinatal mental health support and services for mothers from pregnancy through to the postpartum period and beyond. As such, understanding the broader course of maternal depression is essential for identifying key timepoints for effective intervention and support."
In addition to this restriction, it would be useful to report the number of participants in the following subgroups: A) 0-3 months, B) 4-6 months, C) 7-9 months, and D) 10-12 months. In addition to reporting this, the authors may consider using a histogram for its visualization as well.<br><br>Furthermore, reporting their mean Edinburgh Postnatal Depression Scale (EPDS) score and the prevalence of self-reported depression in these postpartum subgroups would be useful as well. With these results, the authors can	We have specified in Table 1 the sample distribution of the postpartum subgroups according to the number of months postpartum: 0-12 months, 13-24 months, and 25+months. We understand the importance of showing the sample size and prevalence for the subgroups specified by the Reviewer. However, doing so would result in small subgroup sample sizes (small cell sizes), which may lead to low statistical power.<br><br>For transparency, we looked into the prevalence for 0-3 months (n = 26 out of 48, 54.2%, 95% CI = 40.0% - 67.7%), 4-6 months (n = 15 out of 30,

Comment	Response
more appropriately compare their findings from the 0-3 month (0-12 weeks) subgroup to the results seen in the two studies that examined postpartum depression rates in the Philippines (the study samples in these two studies included postpartum persons up to 6 and 8 weeks, respectively).	50.0%, 95% CI = 32.7% - 67.3%), 7-9 months (n = 22 out of 31, 71.0%, 95% CI = 52.8% - 84.3%), and 10-12 months (n = 8 out of 13, 61.5%, 95% CI = 34.1% - 83.2%). These prevalences are not statistically different from those for 0-12 months, 12-24 months, and months, as shown in Table 2.
2) It would also be useful to the reader to report the number of participants in each trimester for the pregnancy sample. In addition to reporting this, the authors may consider using a histogram for its visualization as well.	We have specified in Table 1 the sample distribution of the antenatal subgroups according to trimester.
3) The authors should provide a flowchart describing how many individuals were approached for the study, how many agreed, how many were excluded from the study based on their eligibility criteria, and how many were included in the study sample. The authors should then statistically compare the study sample to those who were excluded based on the eligibility criteria in regards to sociodemographic data. This will determine if their study sample is representative of the study population.	We created the participant selection flowchart for our study, as shown in Figure S1, and included it as part of the supplemental file.<br><br>We did not collect the socio-demographic information of those who were excluded due to ineligibility. However, we were able to compare our sample distribution to recent perinatal statistics in the Philippines (Philippine Statistics Authority, 2024; Philippine Statistics Authority and ICF, 2023). As shown in Table S4, our sample is generally comparable with the national statistics in terms of age, marital status, educational attainment, first pregnancy status, and prenatal visit attendance. While this is the case, the results from our study should be interpreted with caution as our sample is not representative of the whole country. We specified this in the Discussion>Limitation section (page 16).<br><br>"...While our sample is comparable to the Philippines' perinatal national statistics (Table S4),[35] [36] results from the survey should be interpreted with caution as our sample is not country-representative..."
4) If the authors have access to data on the State-Trait Anxiety Inventory Scale, Perceived Stress Scale-4, and De Jon Gierveld Loneliness Scale, they should consider reporting these scores as well in their study. The COCOON Global Collaboration protocol (which the current study is apart of) used these scales in addition to the EPDS. The level of anxiety, stress, and social loneliness among participants may help explain the alarmingly high rates of self-reported depression in pregnancy and the postpartum within this study. If present, the authors should report	We have collected data on the State-Trait Anxiety Inventory (STAI), Perceived Stress Scale-4 (PSS-4), and the De Jong Gierveld Loneliness Scale as part of the COCOON Global Collaborative Study. However, the PSS-4 demonstrated poor reliability in the Philippine sample in this present study (Cronbach's alpha: pregnant sample = 0.2048; postpartum sample = 0.1383). Therefore, we decided not to further analyse the data from this scale.<br><br>While we recognise the importance of examining anxiety, stress, and social loneliness in

Comment	Response
these scores and statistically compare them among those with self-reported depression and those without self-reported depression.	understanding depressive symptoms, the analyses and reporting of findings from these scales will be presented separately, in accordance with the publication plan of the COCOON Global Collaboration.
5) The authors should consider reporting that a limitation of their study is the lack of data on psychiatric (e.g., personal history of mental disorders) and obstetric (e.g., gestational complications) factors in their study. There could be differences in these factors among those with self-reported depression and those without self-reported depression.	We recognised this limitation in the Discussion>Limitations section (page 16) as suggested.<br><br>"…Second, it could be argued that online-based recruitment introduced sampling bias, potentially resulting in a skewed sample of mothers with existing mental health problems. However, we did not observe significant differences in item-level responses on the EPDS between those who were recruited online and face-to-face. Although we did not collect comprehensive data on formal psychiatric diagnoses, we asked participants about their use of mental health services and medications. Only two postpartum women reported receiving psychological therapy or counselling, and four reported current use of psychiatric medications. These findings suggest a low likelihood of sampling bias related to mental health status…"
6) Lines 15 to 19 on page 14 in the Discussion needs to be removed as the authors did not examine this in their study based on their Methodology and Results sections.	These sentences have been removed as per the Reviewer's suggestion.
7) Minor revisions: A) Please report the sample size for the whole study sample as well as the pregnancy and postpartum subsamples in the Findings section of the Abstract;	The sample of the whole study and sub-samples have been added.<br><br>"**Results:** A total of 856 women participated in the study, comprising 356 pregnant and 500 postpartum women…"
B) Please report the sample size for reference 7 in lines 21-22 on page 14 of the Discussion;	We have included the sample size and recruitment details for reference 7 in the Discussion section (page 14).<br><br>"…For example, a pre-pandemic survey of 165 mothers who accessed maternal care from five rural health facilities showed lower postpartum depression prevalence estimates of 16.4% using an EPDS cut-off of ≥10.[8]"
C) Please report the prevalence rate reported for reference 15 in lines 36-40 on page 14 of the Discussion.	We have revised the Discussion section (page 14) to include the specific pooled prevalence rates for

Comment	Response
	antenatal and postpartum depression reported in reference 15 (now reference 22).<br><br>"...The similarity in prevalence between these groups aligns with findings from previous research, including an umbrella review of 12 meta-analyses, which reported comparable global prevalence of depressive symptoms across the perinatal period during the pandemic (antenatal depression = 29.0% and postpartum depression = 26%.0).[22] However, the prevalence estimates found by our study are alarmingly higher compared to the global prevalence pooled by his umbrella review and the pre-pandemic prevalence in another review in Southeast Asia.[23]..."

Reviewer 3

Comment	Response
General comment<br>I would like to congratulate the authors on conducting a much-needed research project on the prevalence of perinatal depression in the Philippines. The article is well written, with clearly described methods and results. I only have a few minor comments that I believe could help improve the clarity of the methodology and better contextualize the findings.	We thank the Reviewer for this feedback. We have outlined our revisions to the manuscript in the following responses.
Background / Discussion<br>• Consider including	We added general information about the mental health status of women in the Philippines in the Introduction section (page 5).

Comment	Response
a broader discussion of mental health conditions among Filipino women, either in the background or discussion section. This would provide important context, especially given the alarmingly high prevalence rates reported during pregnancy and postpartum.	"...The mental health burden among Filipino women remains poorly documented, and studies on perinatal mental health are particularly scarce. The only national data on women's mental health comes from the 2021 Philippine National Survey on Mental Health and Well-being. This survey showed that the lifetime and past 12-month prevalence of any mental disorder among women aged 18 years or older were 12.7% and 9.63%, respectively.[5] The survey also indicated that 1.61% and 1.66% of women experienced major depressive disorder and a major depressive episode in the past 12 months..."
Methods<br>• The description of women with miscarriage, stillbirth, or neonatal death appears inconsistent. Were these women excluded from the analysis? If so, why is it stated in the discussion that their small number would not have substantially affected the results?	We apologise for the confusion and have removed this point from the Discussion>Limitations section. As outlined in our published protocol (Loughnan et al., 2022), we have a dedicated survey for mothers who have experienced stillbirth or neonatal death. Due to this stratified sampling approach, merging this group with the general postpartum sample could lead to an overestimation of results. In line with the publication plan of the COCOON Global Collaboration, findings relating to mothers who experienced perinatal loss are being reported separately.
• Please indicate how many maternal care facilities were involved in recruitment, and from which geographical regions.	Face-to-face recruitment was conducted in a total of seven maternal care facilities across Metro Manila and Rizal Province. This was reflected in the Methods section (page 6).<br>"...We increased the reach of recruitment by approaching eligible pregnant and postpartum women face-to-face in seven maternal care facilities, including four community health centres and three birthing clinics across Metro Manila and Rizal Province, from November 2023 to May 2024."
• The manuscript states that "an internal consistency" was calculated for the EPDS during pregnancy and	We added the Cronbach's alpha of the EPDS based on our sample to the Methods section (page 7).<br>"...In this study, EPDS has a Cronbach's alpha of 0.851 among pregnant women and 0.860 among postpartum women..."

Comment	Response
postpartum, but no details of the statistical method are provided. Please specify the type of reliability coefficient used (e.g., Cronbach's alpha).	
• On page 6, lines 33–35, the authors describe verifying participants' names, gestational age, and delivery date. How was this verification conducted? Which databases or administrative records were accessed?	We verified participants' details based on their self-reports during our initial conversations via Facebook Messenger, as we do not have access to any third-party databases or administrative records. The primary purpose of this verification process was to determine whether the accounts were genuine and not operated by bots or fraudulent users. For instance, some participants used special characters in their account names, prompting us to confirm that they were real individuals. In one case, we identified a prospective participant using their deceased daughter's account. Upon verification, we informed the user of the outcome and ceased further contact.<br><br>We also checked whether participants met our eligibility criteria, including sex (i.e., female), date of delivery, and gestational age. Additionally, we verified that their reported date of birth aligned with their self-reported age. We discontinued contact with users who provided inconsistent or misleading information.
• A flowchart would greatly improve transparency. On page 8, line 17, it is stated that "all participants responded to all EPDS items," whereas line 46 indicates that only responses with at least one completed EPDS were included. A flow diagram would clarify exclusions due to missing EPDS data.	To clarify, none were excluded from the final sample due to non-completion of the whole EPDS module. This means all the participants completed all 10 items of the EPDS.<br><br>The final sample included participants who had completed at least the Psychosocial Health module, which contains the EPDS. Because this module appears in the latter part of the questionnaire, participants who partially completed the survey up to this point had only skipped the socio-demographic set of questions. The number of participants who fully and partially completed the survey is reflected in the participant selection flowchart for our study (Figure S1).
• Has the EPDS been validated in the Philippines? If not, are there validation studies or cutoff	The most recent diagnostic validation in the Philippines was conducted in 2010 (Balce-Mercado, 2011). This study suggested a cut-off of ≥13 with 84% sensitivity and 85% specificity. We have now used this as our cut-off score for prevalence estimation and regression analysis.

Comment	Response
recommendations from culturally comparable populations? This is particularly relevant given the high prevalence estimates reported.	
• Please clarify how recruitment via social media was conducted. Is it possible that women experiencing mental health difficulties were more likely to respond to such outreach, which could partially explain the high prevalence?	Recruitment through social media involved paid Facebook advertisements deployed weekly from March to April 2024. Interested users contacted the research team through Facebook Messenger, where eligibility screening and verification were carried out. While we acknowledge the potential for sampling bias, further analysis of individual EPDS item responses revealed no statistically significant differences between participants recruited online and those recruited face-to-face.<br><br>We found no statistical difference in response patterns between modes of recruitment except for item 10 (p=0.034) among pregnant women, and items 2 (p=0.046), item 8 (p=0.036), and item 10 (p=0.017) among postpartum women. Higher reports of self-harm behaviour (item 10) among online participants (pregnant: 33.7% vs 23.7%; postpartum: 34.4% vs 22.0%) are expected due to the known stigma on this behaviour. This finding was reflected in the Discussion section (page 16).<br><br>"...Second, it could be argued that online-based recruitment introduced sampling bias, potentially resulting in a skewed sample of mothers with existing mental health problems. However, we did not observe significant differences in item-level responses on the EPDS between those who were recruited online and face-to-face..."
Results<br>• The finding that "most pregnant women had previous pregnancies (68.1%), while postpartum women were primarily experiencing their first pregnancy (69.5%)" appears contradictory. This raises concerns about how parity was assessed. It is conceivable that postpartum women excluded their	We apologise for this confusion. This variable pertains to whether the reference pregnancy was their first pregnancy or not. We have revised this text along with the labels in the tables to clarify this variable.

Comment	Response
current pregnancy from the count—if so, please clarify the wording of this question.	
• In Table 1, some percentage values appear to be missing. Please ensure all percentage values are presented unless truly zero.	Table 1 has now been checked to ensure no cells have missing percentages.
Discussion<br>• Please elaborate on the generalizability of the findings. Are there national statistics on perinatal characteristics such as maternal age, gestational age at delivery, or relationship status? If so, including or citing these would strengthen the paper by demonstrating how representative the study population is.	We thank the Reviewer for this suggestion. We were able to compare our sample distribution to recent perinatal statistics in the Philippines (Philippine Statistics Authority, 2024; Philippine Statistics Authority and ICF, 2023), as shown in Table S4. We found that our sample is generally comparable with the national statistics in terms of age, marital status, educational attainment, first pregnancy status, and prenatal visit attendance. While this is the case, the results from our study should be interpreted with caution as our sample is not representative of the whole country. We specified this in the Discussion>Limitation section (page 16).<br><br>"...While our sample is comparable to the Philippines' perinatal national statistics (Table S4),[35 36] results from the survey should be interpreted with caution as our sample is not country-representative..."
• Throughout the manuscript, consider replacing the term depression with depressive symptoms, as the EPDS is a screening tool rather than a diagnostic instrument.	We have replaced "depression" with "depressive symptoms" or "clinically significant depressive symptoms" whenever appropriate. To also ease the discussion of our results and implications, we also specified in the Methods section (page 8):<br><br>"...The prevalence of clinically significant perinatal depressive symptoms (also referred to as perinatal depression in this paper)..."

Comment	Response
• The potential influence of the COVID-19 pandemic on the results should be discussed more explicitly. Could elevated symptom levels reflect pandemic-related stressors? Expand the comparison with previous studies from the Philippines. What factors might explain the discrepancy in prevalence—differences in setting, timing (pre- vs post-pandemic), population characteristics, or methodology?	The Reviewer's feedback is noted, as this aspect deserves further attention. To address this, we revised the Discussion section (pages 14-15) to include findings from relevant studies.<br><br>"The differences in prevalence between our study and previous research may be explained by the profound impact of the pandemic on women's mental health as well as the persistent gaps in perinatal mental health services in the Philippines. The Global Burden of Disease study reported a disproportionate rise in major depressive disorder (MDD) prevalence among women compared to men, with the Philippines experiencing the highest increase in MDD prevalence across Southeast Asia.[24] A recent meta-analysis of 22 studies comparing postpartum depression prevalence before and during the pandemic found a significant increase in prevalence regardless of perinatal time period.[25] This prevalence increase was only observed in Asian countries particularly in China, Japan and Israel. Notably, this paper reported no significant difference in prevalence changes between developed and developing countries.<br><br>In the Philippines, no policy currently supports routine mental health screening and support among perinatal populations, despite its successful implementation of maternal health programs.[26 27] This calls for the public health system in the Philippines to reinforce the implementation of its Mental Health Act[28] to improve the provision of basic and specialised services for pregnant and postpartum women. Our research provides empirical support for the World Health Organization's recommendations on perinatal mental health integration with maternal and child health services.[29] The consistently high prevalence of depression across the perinatal period implies a universal approach in early detection, low-intensity interventions, and referral to specialised care. An extended surveillance beyond the conventional 12-month postpartum period would also benefit mothers who delayed seeking care.<br><br>We found no statistically significant differences in depressive symptoms across pregnancy trimesters or months since childbirth, suggesting a persistently elevated risk throughout the perinatal period. Although perinatal depressive symptoms are generally expected to improve over time, recent evidence indicates that the likelihood of experiencing persistently high or worsening symptoms over the 36 months postpartum is comparable to the likelihood of following an improving mental health trajectory.[14 15] A cross-country cohort investigation of 11,563 women in the UK, Canada and Singapore showed consistent clinical symptom levels of depression from pregnancy up to 24 months postpartum.[16] A systematic review of 21 studies from six LMICs and 15 HICs also concluded that maternal depression remained prevalent beyond the first year postpartum, particularly in marginalised groups.[17] Although depressive symptoms beyond 12 months postpartum may be influenced by non-perinatal factors, persistent depressive symptoms may also be attributed to poor help-seeking behaviour and the limited availability of perinatal mental health support and services for mothers from pregnancy through to the

Comment	Response
	postpartum period and beyond. As such, understanding the broader course of maternal depression is essential for identifying key timepoints for effective intervention and support.”

References

Balce-Mercado, D. J. (2011). Screening postpartum depression with the Filipino version of the Edinburgh postnatal depression scale. *Journal of the Perinatal Association of the Philippines*, *15*(1). https://www.herdin.ph/index.php?view=research&cid=56371

Hunter, T. R., Chiew, B. A., McDonald, S., & Adhikari, K. (2024). The Prevalence of maternal depression and anxiety beyond 1 year postpartum: A systematic review. *Maternal and Child Health Journal*, *28*(8), 1283-1307. https://doi.org/10.1007/s10995-024-03930-6

Kee, M. Z. L., Cremaschi, A., De Iorio, M., Chen, H., Montreuil, T., Nguyen, T. V., Côté, S. M., O’Donnell, K. J., Giesbrecht, G. F., Letourneau, N., Chan, S. Y., & Meaney, M. J. (2023). Perinatal trajectories of maternal depressive symptoms in prospective, community-based cohorts across 3 continents. *JAMA Network Open*, *6*(10), e2339942-e2339942. https://doi.org/10.1001/jamanetworkopen.2023.39942

Kemp, S. (2025). *Digital 2025: The Philippines*. DataReportal. Retrieved 17 October 2025 from https://datareportal.com/reports/digital-2025-philippines

Loughnan, S. A., Gautam, R., Silverio, S. A., Boyle, F. M., Cassidy, J., Ellwood, D., Homer, C., Horey, D., Leisher, S. H., de Montigny, F., Murphy, M., O'Donoghue, K., Quigley, P., Ravaldi, C., Sandall, J., Storey, C., Vannacci, A., Wilson, A. N., & Flenady, V. (2022). Multicountry study protocol of COCOON: COntinuing Care in COVID-19 Outbreak global survey of New, expectant, and bereaved parent experiences. *BMJ Open*, *12*(9), e061550. https://doi.org/10.1136/bmjopen-2022-061550

Philippine Statistics Authority. (2024). *Registered live births in the Philippines, 2023* https://psa.gov.ph/content/registered-live-births-philippines-2023

Philippine Statistics Authority and ICF. (2023). *2022 Philippine National Demographic and Health Survey (NDHS): Final report*. https://www.dhsprogram.com/pubs/pdf/FR381/FR381.pdf

Putnick, D. L., Sundaram, R., Bell, E. M., Ghassabian, A., Goldstein, R. B., Robinson, S. L., Vafai, Y., Gilman, S. E., & Yeung, E. (2020). Trajectories of maternal postpartum depressive symptoms. *Pediatrics*, *146*(5). https://doi.org/10.1542/peds.2020-0857

VERSION 2 - REVIEW

**Reviewer**	**1**
**Name**	**Matsumura, Kenta**
**Affiliation**	**University of Toyama, Public Health**
**Date**	**01-Nov-2025**
**COI**	

I commend the authors' efforts. They have responded appropriately to all of my comments. Thank you.

**Reviewer**	**2**
**Name**	**Dama, Manish**
**Affiliation**	**Trinity College Dublin School of Medicine**
**Date**	**07-Nov-2025**
**COI**	

I would like to thank the authors for their revisions to this manuscript. Despite the changes that they have made, unfortunately, I am recommending a rejection of the manuscript in its revised form.

The authors may want to consider resubmitting their manuscript as a brief report/communication to BMJ open or another journal, as their findings about the prevalence rate of antenatal depression in the Philippines is important. This evidence will help in development of health policies to support the care of perinatal individuals in the Philippines.

As for the revised manuscript, I have recommended a rejection for the following reasons:

1) I do agree with the authors that perinatal depressive symptoms can persist beyond 12 months postpartum for some perinatal individuals and the authors did provide some evidence to support this: 1) Putnick et al. 2020 and 2) Kee et al. 2023.

However, this current study utilizes a survey study design (i.e., a cross-sectional study design) that examined depressive symptoms only once. Among the 500 postpartum individuals in their sample, 371 (74.2%) of them were assessed for depressive symptoms 13 months or more beyond childbirth. Therefore, the authors are unable to determine whether these 371 individuals were experiencing depression between pregnancy and 12-months postpartum (i.e., the perinatal period).

As described in my initial review of the current manuscript, it is possible that some of the participants who are experiencing self-reported depression after 12 months postpartum may have developed depressive symptoms due to non-perinatal reasons. Without a longitudinal study design, the authors of the current manuscript cannot conclude that the depressive symptoms that develop during the perinatal period persisted beyond 12 months postpartum, nor that those who developed depressive symptoms after 12 months postpartum is due to perinatal factors.

The Putnick et al 2020 and Kee et al 2023 studies utilized prospective cohort study designs that followed their participants from pregnancy to beyond 12-months postpartum and

assessed the symptoms of the same individuals at multiple points throughout pregnancy and beyond 12-months postpartum. Thus, these two past studies have demonstrated that the depressive symptoms that have developed during the perinatal period can persist beyond 12-months postpartum.

Furthermore, the authors also provided the citation of a systematic review conducted by Hunter et al 2024 as well to support their revision. However, in the exclusion criteria of this systematic review, they excluded studies of participants at or before 1-year postpartum. This means that this systematic review was interested in examining maternal depression and anxiety that developed after the perinatal period (i.e., non-perinatal depression and anxiety). Therefore, this citation does not support their revision.

2) The authors did not include the recommendation of examining the prevalence of postpartum depression at 0-3 months, 4-6 months, 7-9 months, and 10-12 months into their manuscript. I understand that the subgroup sample sizes are small, but these could have been placed in the supplementary materials, and their 0-3 months subsample (N = 48) is comparable to Garcia et al 2024 (N = 61; one of the two studies that examined the prevalence rate of postpartum depression in the Philippines). This would have allowed a more appropriate comparison of their work with Garcia et al 2024's findings.

3) The authors chose to not incorporate the findings of the STAI, PSS-4, or the De Jong Gierveld Loneliness Scale into their work, despite having the results. I understand that the PSS-4 demonstrated poor reliability, but the authors could have included the STAI and De Jong Gierveld Loneliness Scale into their study. I recommended this in the initial review to help the authors explain why the rate of perinatal depression is so high in their sample (e.g., is a high degree of social loneliness linked to elevated levels of depression among perinatal individuals in the Philippines?).

Overall, I believe this revised manuscript should be repackaged into a brief communication/report for BMJ Open or another journal. I hope the 3 reasons described above will help them in the repackaging of their manuscript.

**Reviewer**	**3**
**Name**	**Papadopoulos, Fotios**
**Affiliation**	**Uppsala Univ, Department of Medical Sciences, Psychiatry**
**Date**	**23-Nov-2025**
**COI**	

Thank you for the thoughtful revision of this paper, which is much improved. I have only two minor comments:

- In page 11 : The authors write about “Perinatal depressive symptoms” that “ The mean EPDS score was similar between pregnant (Mean=14.4, SD=5.7, range=0-29) and postpartum women (Mean=14.1, SD=6.2, range=0-30). “

However, this is not in line with the last raw of Table 1, where the mean EPDS during pregnancy was 13.6 and postpartum 13.7.

- Table 2: clarify the time effect definition.

In the methods the paragraph about time is not easy to follow “Regression analyses were used to examine the trend of depressive symptoms and prevalence by perinatal time period and by timing of birth (among postpartum women). The timing of birth was categorised into two groups: during the pandemic and after the pandemic (from July 2023 onward), reflecting the lifting of the COVID-19 public health emergency status by the Philippine government in July 2023.21 Analysis by perinatal time period was performed using the combined pregnant and postpartum sample, with a time variable generated based on AOG among pregnant women and months after birth among postpartum women (time variable categories: 1st, 2nd, and 3rd trimesters, 0-12 months, 13-24 months, and 25+ months postpartum). “

In this paragraph the timing is defined as : 1) pandemic related, 2) 1st, 2nd, and 3rd trimesters, 0-12 months, 13-24 months, and 25+ months postpartum.

Maybe add the results for the pandemic in the same table?

Start with Time since pregnancy start (1st trimester, 2nd trimester…. 25+ months postpartum)

And

Continue with Birth during the pandemic and Birth after the pandemic.

VERSION 2 - AUTHOR RESPONSE

Reviewer 1

Comment	Response
I commend the authors' efforts. They have responded appropriately to all of my comments. Thank you.	We thank the Reviewer for this feedback.

Reviewer 2

Comment	Response
1) I do agree with the authors that perinatal depressive symptoms can persist beyond 12 months postpartum for some perinatal individuals and the authors did provide some evidence to support this: 1) Putnick et al. 2020 and 2) Kee et al. 2023.<br><br>However, this current study utilizes a survey study design (i.e., a cross-sectional study design) that examined depressive symptoms only once. Among the 500 postpartum individuals in their sample, 371 (74.2%) of them were assessed for depressive symptoms 13 months or more beyond childbirth. Therefore, the authors are unable to determine whether these 371 individuals were experiencing depression between pregnancy and 12-months postpartum (i.e., the perinatal period).<br><br>As described in my initial review of the current manuscript, it is possible that some of the participants who are experiencing self-reported depression after 12 months postpartum may have developed depressive symptoms due to non-perinatal reasons. Without a longitudinal study design, the authors of the current manuscript cannot conclude that the depressive symptoms that develop during the perinatal period persisted beyond 12 months postpartum, nor that those who developed depressive symptoms after 12 months postpartum is due to perinatal factors.<br><br>The Putnick et al 2020 and Kee et al 2023 studies utilized prospective cohort study designs that followed their participants from pregnancy to beyond 12-months postpartum and assessed the symptoms of the same individuals at multiple points throughout pregnancy and beyond 12-months postpartum. Thus, these two past studies have demonstrated that the depressive symptoms that have developed during the perinatal period can persist beyond 12-months postpartum.<br><br>Furthermore, the authors also provided the citation of a systematic review conducted by Hunter et al 2024 as well to support their revision. However, in the exclusion criteria of this systematic review, they excluded studies of participants at or before 1-year postpartum. This means that this systematic review was interested in examining maternal depression and anxiety that developed after the perinatal period (i.e.,	We acknowledge the reviewer's point regarding the limitations of a cross-sectional design in determining the timing of depressive symptom onset. Accordingly, we have revised the relevant sentence in the Discussion (page 15) by removing the statement that implied our results demonstrated symptom persistence throughout the perinatal period.<br>"We found no statistically significant differences in depressive symptoms and prevalence across pregnancy trimesters or months since childbirth~~, suggesting a persistently elevated risk throughout the perinatal period~~."<br><br>We cited the longitudinal studies by Putnick et al. (2020) and Kee et al. (2023) because their prospective designs provide robust evidence that depressive symptoms emerging during the perinatal period may persist beyond 12 months postpartum. While our study cannot assess symptom trajectories, these longitudinal findings offer relevant context for interpreting elevated depressive symptom prevalence among participants assessed beyond 12 months postpartum. Previous evidence has also used 24 months as the upper limit for assessing perinatal depression. Hence, we cited Hunter et al. (2024) to demonstrate consistency of prevalence within the 24-month mark and beyond. Citation of other cross-sectional studies would not address this temporal limitation.<br><br>Considering the variability in defining the perinatal period, we concluded by emphasising the need for a comprehensive understanding of perinatal depression and its contributing factors across time, see Discussion section (page 15):<br>"Although depressive symptoms beyond 12 months postpartum may be influenced by non-perinatal factors, persistent

Comment	Response
non-perinatal depression and anxiety). Therefore, this citation does not support their revision.	depressive symptoms may also be attributed to poor help-seeking behaviour and the limited availability of perinatal mental health support and services for mothers from pregnancy through to the postpartum period and beyond. As such, understanding the broader course of maternal depression is essential for identifying key timepoints for effective intervention and support."<br><br>It is not feasible to identify a highly comparable cross-sectional study with an extended assessment period to further support our findings, due to the limited local and regional literature. To identify relevant local evidence, the lead authors (JAF and JCM) conducted an electronic search using PubMed, Scopus, Google Scholar, and HERDIN with the terms: (perinatal OR antenatal OR pregnancy OR postnatal OR postpartum) AND (depression OR "depressive symptoms") AND (Philippines OR Filipino*). Evidence remains scarce: only three small facility-based studies have examined postpartum depression prevalence before (n=2) or during (n=1) the pandemic. A global meta-analysis of perinatal depression in low- and middle-income countries revealed substantial country-level variation and poor representation from the Philippines (n=1) and neighbouring countries (Cambodia: n=0; Laos: n=1; Myanmar: n=0; Timor-Leste: n=2).<br><br>We note that this study provides novel, population-level data on postpartum depressive symptoms in the Philippines, where perinatal mental health research, particularly large-scale and community-based studies, remains limited. We hope that these findings contribute foundational evidence to inform future longitudinal research and strengthen perinatal mental health policy and services in this setting.
2) The authors did not include the recommendation of examining the prevalence of postpartum depression at 0-3 months, 4-6 months, 7-9 months, and 10-12 months into their manuscript. I understand that the	We have added a supplementary table, Table S4, which shows the prevalence and mean EPDS score of women who gave birth 12 months prior to the survey. We

Comment	Response
subgroup sample sizes are small, but these could have been placed in the supplementary materials, and their 0-3 months subsample (N = 48) is comparable to Garcia et al 2024 (N = 61; one of the two studies that examined the prevalence rate of postpartum depression in the Philippines). This would have allowed a more appropriate comparison of their work with Garcia et al 2024's findings.	disaggregate the results according to the Reviewer's recommendation. We have also revised the appropriate text in the Discussion where we compared our results with Garcia et al, 2024's findings, see Discussion section (page 15):<br>"A hospital-based study of 61 postpartum women who gave birth during the pandemic also showed a lower prevalence of 32.8% (versus 54.2% found by our study among mothers 0-3 months postpartum, see Table S4) despite using a higher score threshold (EPDS score ≥13)."
3) The authors chose to not incorporate the findings of the STAI, PSS-4, or the De Jong Gierveld Loneliness Scale into their work, despite having the results. I understand that the PSS-4 demonstrated poor reliability, but the authors could have included the STAI and De Jong Gierveld Loneliness Scale into their study. I recommended this in the initial review to help the authors explain why the rate of perinatal depression is so high in their sample (e.g., is a high degree of social loneliness linked to elevated levels of depression among perinatal individuals in the Philippines?).	We appreciate the reviewer's thoughtful comment and the suggestion to consider the STAI and De Jong Gierveld Loneliness Scale data.<br><br>Our primary aim in this paper is to present the prevalence of perinatal depression and patterns across socio-economic groups, as outlined in the Introduction. As a foundational study on perinatal depression in the Philippines, we sought to provide streamlined results with clear and actionable implications for policy and program development.<br><br>While we recognise that measures of anxiety and loneliness can offer important contextual insights, we believe that exploring these psychosocial factors warrants a more in-depth investigation than is feasible within the scope of the present paper. For example, emerging evidence suggests that loneliness may mediate the relationship between socio-cultural factors and maternal depression. In addition, examining correlations with anxiety symptoms would add limited contribution, given the well-established co-occurrence of anxiety and depressive symptoms in the literature.

Reviewer 3

Comment	Response
Thank you for the thoughtful revision of this paper, which is much improved. I have only two minor comments:<br><br>- In page 11 : The authors write about "Perinatal depressive symptoms" that " The mean EPDS score was similar between pregnant (Mean=14.4, SD=5.7, range=0-29) and postpartum women (Mean=14.1, SD=6.2, range=0-30). "<br>However, this is not in line with the last raw of Table 1, where the mean EPDS during pregnancy was 13.6 and postpartum 13.7.	We thank the Reviewer for their comments. During our review, we identified a minor coding error that resulted in a slight adjustment to the estimates. Importantly, this correction did not affect the interpretation of our results, the findings from the regression analyses, or the comparative discussions with previous studies.
- Table 2: clarify the time effect definition.<br>In the methods the paragraph about time is not easy to follow. In this paragraph the timing is defined as : 1) pandemic related, 2) 1st, 2nd, and 3rd trimesters, 0-12 months, 13-24 months, and 25+ months postpartum.<br><br>Maybe add the results for the pandemic in the same table?<br>Start with Time since pregnancy start (1st trimester, 2nd trimester.... 25+ months postpartum)<br>And<br>Continue with Birth during the pandemic and Birth after the pandemic.	We have revised this paragraph in the Methods section to clarify the definition of time effect used in the analyses.<br>"Regression analyses were used to examine the trend of depressive symptoms and prevalence by perinatal time period (combined pregnant and postpartum sample) and by timing of birth (postpartum women only). Analysis by perinatal time period was performed using a time variable generated based on AOG among pregnant women and months after birth among postpartum women (time variable categories: 1st, 2nd, and 3rd trimesters, 0-12 months, 13-24 months, and 25+ months postpartum). Separate regression analysis was conducted using the timing of birth variable, which has two categories: during the pandemic and after the pandemic (from July 2023 onward). This split reflects the lifting of the COVID-19 public health emergency status by the Philippine government in July 2023.[21] All analyses were adjusted for demographic characteristics and obstetric profile."<br><br>We have also reorganised Table 2 to mirror how the time effect was outlined in the Methods.

VERSION 3 - REVIEW

**Reviewer**	**2**
**Name**	**Dama, Manish**
**Affiliation**	**Trinity College Dublin School of Medicine**
**Date**	**06-Jan-2026**
**COI**	

I would like to thank the authors for their revisions.

I have no additional comments.
